# Chronic Venous Disease in Pregnancy: Pathophysiology, Diagnostic Pitfalls, and a Trimester-Stratified Management Framework—A Narrative Review

**DOI:** 10.3390/jcm15208111

**Published:** 2026-10-19

**Authors:** Raluca Stefana Stan (Bran), Iustina Petra Solomon Condriuc, Raluca Maria Haba, Vasile Adrian Sescu, Raluca Anamaria Mogos, Claudia Florida Costea, Alexandru Carauleanu

**Affiliations:** 1 Grigore T. Popa University of Medicine and Pharmacy, 700115 Iasi, Romaniaraluca.haba@umfiasi.ro (R.M.H.); vasile-adrian.sescu@d.umfiasi.ro (V.A.S.);; 2 Surgical Department, Faculty of Medicine, Grigore T. Popa University of Medicine and Pharmacy, 700115 Iasi, Romania; 3 Department of Obstetrics and Gynecology, Cuza Voda Hospital, 700038 Iasi, Romania; 4 Department of Ophthalmology, Grigore T. Popa University of Medicine and Pharmacy, 700115 Iasi, Romania; 5 Department of Ophthalmology, Prof. Dr. Nicolae Oblu Emergency Clinical Hospital, 700309 Iasi, Romania

**Keywords:** pregnancy, chronic venous disorders, venous reflux, duplex ultrasonography, compression stockings, superficial vein thrombosis, puerperium, evidence extrapolation

## Abstract

Chronic venous disease (CVD) in pregnancy is common, poorly codified, and interpreted with diagnostic thresholds, severity instruments and treatment algorithms all derived outside pregnancy. This narrative review synthesises the mechanistic, epidemiological, diagnostic and therapeutic literature, keeping pregnancy-specific evidence separate from evidence extrapolated from non-pregnant populations. Gestational change is characterised principally by increased venous capacitance and vein-wall distension; ambulatory venous hypertension was not demonstrated in the two pregnancy studies assessing it; and new reflux develops in a minority, undetectable postpartum in the one study reporting it. Physiological adaptation, CVD and thrombotic pathology overlap clinically, deep vein thrombosis and superficial vein thrombosis being at once the principal differential diagnoses and potential complications. Management is correspondingly conservative: compression relieves symptoms but has not been shown to prevent varicose veins, routine initiation of venoactive drugs is not supported, and intervention is deferred other than in exceptional circumstances. A venous finding first detected in pregnancy warrants postpartum reassessment before it is taken for established disease. A trimester-stratified framework spanning preconception to late postpartum reassessment is proposed; it is evidence-informed rather than prospectively validated, and is not a guideline.

## 1. Introduction

Chronic venous disease (CVD) ranks among the commonest conditions of adult life, but how common depends almost entirely on where the diagnostic line is drawn. The Clinical–Etiological–Anatomical–Pathophysiological (CEAP) classification grades clinical severity from telangiectasia and reticular veins (class C1) through varicose veins (C2) and oedema (C3) to skin changes and ulceration (C4–C6). Reported prevalence rises steeply as the lower classes are counted: standardised examination identified trunk varices in roughly a third of adults [1]; the pooled global estimate for class C2 is 19% [2]; general-practice screening reports C1–C6 prevalences of 52–70% by world region once near-universal telangiectasia is included [3]. That definitional spread is the largest obstacle to interpreting the pregnancy literature, in which reported frequencies for varicose veins span 1.13% in administrative coding data [4] to 72.7% when reticular veins and telangiectasia are counted [5].

Against that background, pregnancy occupies an unusual position. Parity is among the commonly reported risk factors for chronic venous disease, both in a global systematic review [2] and in the European guideline [6]. Meta-analysis of nine observational studies (17,109 women) gave an odds ratio of 1.82 (95% CI 1.43–2.33) for varicose veins in ever-pregnant versus never-pregnant women, with considerable heterogeneity (I^2^ = 81%) [7]. Pregnancy is also where venous disease is least well characterised: the Cochrane synthesis of interventions for varicose veins and leg oedema in pregnancy comprises seven randomised trials and 326 women, searched to 31 May 2015 [8]. Pregnancy-specific guidance is correspondingly sparse. The current United Kingdom guideline addresses pregnancy in three short recommendations [9]; the European guideline includes a chapter on special patient characteristics that encompasses pregnant women [6]. Senior phlebologists have named this explicitly as an evidence gap [10].

Three features make the problem clinically demanding rather than merely under-researched. First, physiological and pathological signals overlap almost completely: leg swelling, heaviness, aching, nocturnal cramps and restlessness are expected in advanced gestation [11,12], and the diagnostic thresholds, severity instruments and classification used to interpret them were derived outside pregnancy and have not been re-derived in it. Second, the anatomical substrate producing benign gestational venous change also produces pregnancy-associated deep vein thrombosis. Left-sided predominance, calf asymmetry and iliofemoral involvement characterise thrombosis in pregnancy [13,14], and two of the three components of the only pregnancy-derived clinical decision rule are among them [15,16], so clinical assessment discriminates poorly exactly where misclassification matters most. Third, the therapeutic repertoire contracts sharply. Endovenous and open interventions are not recommended other than in exceptional circumstances [9,17]; sclerotherapy is classified internationally as a relative contraindication in pregnancy, postpartum and lactation [18]; venoactive agents carry labelling restrictions [19,20,21,22]; and non-steroidal anti-inflammatory drugs are to be avoided from 20 weeks [23]. What remains is compression, positional and activity advice, education, and vigilance for complications.

These constraints are not constant across gestation. Plasma volume and cardiac output rise into the third trimester [24,25], iliocaval compression grows more relevant as the uterus enlarges [26], saphenous diameter peaks around 34 weeks [27], superficial vein thrombosis incidence rises through gestation and peaks postpartum [28], and the efficiency of a validated algorithm for suspected pulmonary embolism falls as pregnancy advances [29]. Management that ignores this structure treats a 9-week and a 36-week pregnancy alike.

This review has three aims. The first is to assemble the mechanistic and clinical evidence while keeping pregnancy-specific data visibly separate from extrapolation. The second is to set out the diagnostic errors that the overlap predicts, together with the reasoning that avoids them. The third is to propose a framework organised by gestational epoch rather than by treatment modality, running from preconception to postpartum reassessment and labelling which elements rest on pregnancy data, which on extrapolation and which on convention. What is offered is an evidence-organising synthesis: it arranges positions already present in the cited literature and, where none directly applies, adds a small number of explicitly identified author-derived proposals. The topic itself is not new: a German guideline contains a dedicated pregnancy chapter [17], a working group has published a position paper on pregnancy-associated superficial vein thrombosis [30], and the most developed published decision scheme sets out rules by modality for pregnancy and lactation [31]. What we did not identify was a published framework organising chronic venous disease assessment and management primarily by gestational epoch; the guidance located was predominantly modality-based, thrombosis-centred, or limited to brief pregnancy-specific recommendations. That missing axis is the contribution attempted here.

## 2. Materials and Methods

### 2.1. Design and Reporting

This narrative review used a targeted literature search directed at the clinical and mechanistic questions defined in Section 1. The evidence identified was synthesised narratively and interpreted critically, rather than subjected to formal study-level risk-of-bias grading or quantitative pooling. The six items of the Scale for the Assessment of Narrative Review Articles (SANRA) [32] were used as a checklist during drafting and methodological review, and Supplementary Table S5 shows where each item is addressed. SANRA appraises narrative review articles as a whole; it is not a study-level risk-of-bias instrument and does not grade the certainty of the evidence reviewed. Evidence provenance is labelled as described in Section 2.4. The reasons for preferring a narrative synthesis to a systematic or scoping review, and the limitations that choice imposes, are set out in Section 9.

### 2.2. Literature Search

PubMed/MEDLINE and Europe PMC were searched, and the Cochrane Library, the NICE evidence repository and the websites of guidance-issuing bodies, including ESVS, SVS/AVF/AVLS, RCOG, ACOG, ASH, ESC, SOMANZ, AWMF, EMA and FDA, were consulted; Crossref and OpenAlex were used for record lookups. Searching began in January 2026 and was last updated on 20 August 2026. No lower date limit and no language restriction were applied. Four concepts were combined: venous disease, including superficial and deep vein thrombosis and vulvar and pelvic varices; pregnancy and the puerperium; assessment; and management. Searches were directed at the questions addressed in Section 3, Section 4, Section 5, Section 6, Section 7 and Section 8, and reference lists of retrieved articles were checked for additional relevant sources. The principal search terms for each concept, with an illustrative example of how the concepts were combined, are given in Supplementary Text S1.

### 2.3. Scope and Types of Literature Included

The review concerns the venous system, its assessment and its management in pregnancy and the puerperium. Pregnant women are the population of interest throughout. Postpartum women are included where the question concerns reversibility, persistence, thrombotic risk or management after delivery. Evidence from non-pregnant populations is used where no pregnancy-specific source addressing the same question was identified, and is identified as extrapolated at the point of use. The conditions covered are chronic venous disease and its classification, pregnancy-related venous remodelling, varicose veins and reflux, oedema insofar as it bears on venous assessment, vulvar and pelvic venous disease, and superficial vein thrombosis, together with the thrombotic differential diagnoses that cannot be separated from chronic venous disease at the bedside. Venous thromboembolism is considered only as far as diagnostic safety and the boundary decisions of Section 6.2 require; this is not a review of thromboembolism in pregnancy.

The literature considered comprised guidelines, consensus statements and regulatory or product documents; systematic reviews and meta-analyses; randomised trials; observational, diagnostic-accuracy and physiological studies; and seminal mechanistic work. Case series and individual case reports were used where no higher-level pregnancy evidence was identified, as with the published experience of very large vulvovaginal varicosities (Section 7). The outcomes of interest were venous diameter and capacitance, reflux, venous pressure and lower-limb haemodynamics, symptoms and oedema, diagnostic performance and thrombotic outcomes, treatment response and safety, and postpartum persistence or regression. Sources in which venous findings were incidental were not used, and where a later and more complete source addressed the same question, it was preferred. Sources were selected for their relevance to the questions set out in Section 1 and weighted as described in Section 2.4.

The authors’ contributions to the development of the review included methodology (R.S.S., I.P.S.C. and R.A.M.), investigation (R.S.S., R.M.H., V.A.S. and R.A.M.), data curation (R.S.S. and R.A.M.) and validation (I.P.S.C., R.M.H., V.A.S., C.F.C. and A.C.), as detailed in the Author Contributions statement. Literature identification and selection were conducted within the targeted, question-directed narrative process described in Section 2.1, Section 2.2 and Section 2.3.

### 2.4. Source Prioritisation and Evidence Provenance

Sources were prioritised in the following order: current guidelines and consensus documents; systematic reviews and meta-analyses; prospective studies in pregnant women; large observational cohorts; mechanistic studies in pregnant humans; and smaller but clinically informative studies. This ordering determined which source was preferred when several addressed the same question; it does not by itself indicate greater certainty, methodological quality or applicability to pregnancy. A randomised trial conducted outside pregnancy was therefore not treated as stronger evidence about pregnancy than a small physiological study in pregnant women. Within every tier, evidence generated in pregnant or postpartum women took precedence over evidence of equal or higher design quality generated outside pregnancy; a non-pregnant source was used only where no pregnancy-specific source addressing the same question was identified, and is named as extrapolated at the point of use. Emphasis was placed on literature from approximately the last fifteen years, with seminal older work retained where it remains the primary source for a physiological observation, as with the classic venographic studies of the inferior vena cava [26] and early plethysmographic studies of venous distensibility [33].

**Figure 1:**
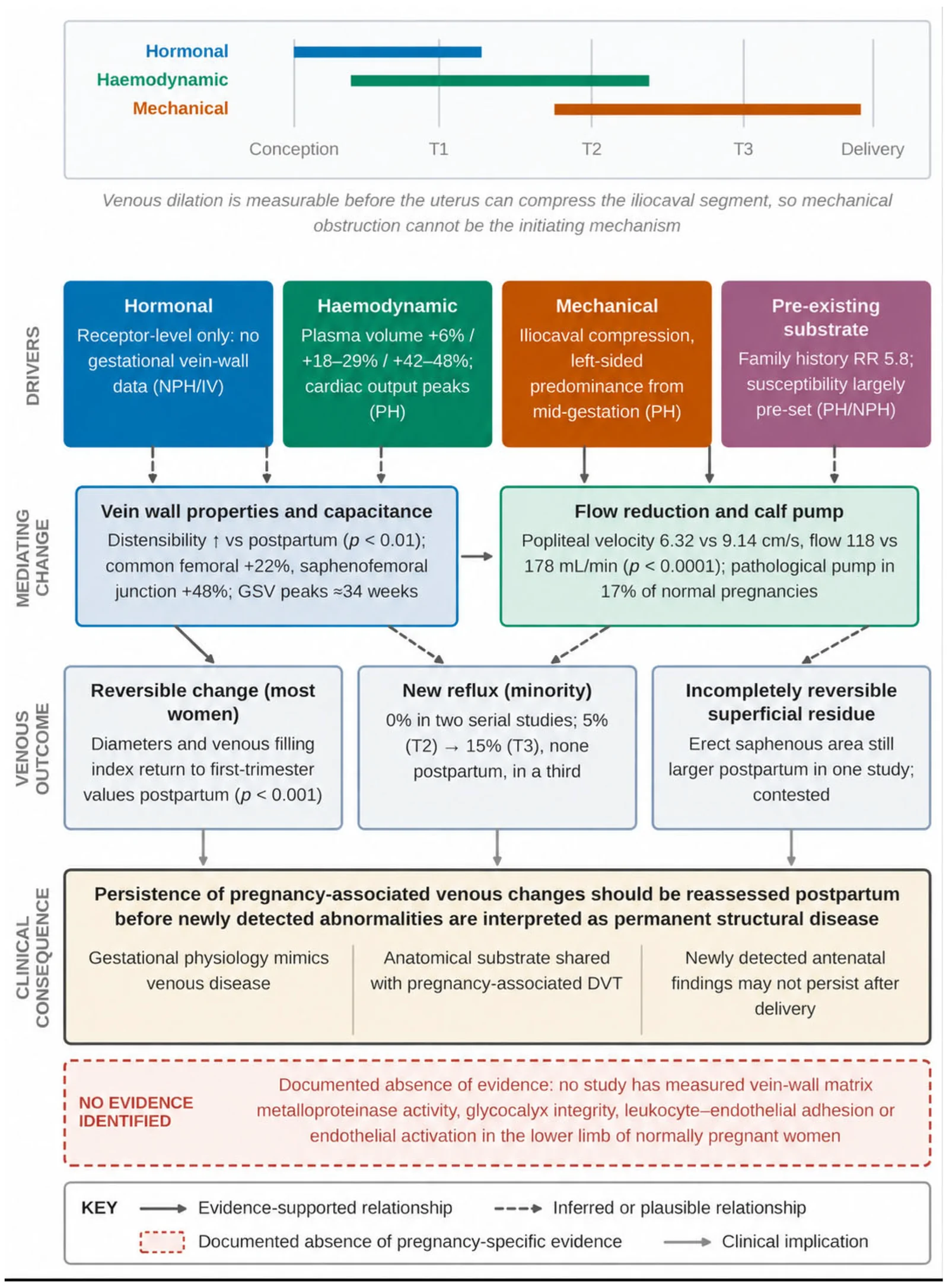
Integrated pathophysiological schema of the venous system in pregnancy. Four upstream drivers act through two groups of mediating change to produce three venous outcomes. Solid arrows indicate evidence-supported or directly represented relationships, without implying demonstrated causality, and dashed arrows inferred ones, including mechanistically plausible but unconfirmed relationships; grey arrows indicate clinical implications of the venous outcomes. All three arrow types are defined in the key within the figure. The timeline shows when the hormonal, haemodynamic and mechanical drivers become dominant, each dominance interval being labelled as well as colour-coded. The dashed panel marks a documented absence of pregnancy-specific evidence. Quantitative values and their sources are given in Table S1. DVT, deep vein thrombosis; GSV, great saphenous vein; IV, in vitro; NPH, non-pregnant human; PH, pregnant human; RR, relative risk; T1, T2, T3, first, second and third trimesters.

Two labelling conventions apply throughout, and they are distinct. The first records where an observation was generated: PH, pregnant human; NPH, non-pregnant human; A, animal; IV, in vitro. These labels are used in Figure 1 and Supplementary Table S1, and the same distinction is carried in words in the narrative text. The second records how a recommendation is supported: [PS], pregnancy-specific, resting on data generated in pregnant or postpartum women; [EX], extrapolated, resting only on data generated outside pregnancy; [CV], convention, where no directly applicable data were identified and the position rests on guideline convention or expert opinion. These labels are used in Supplementary Tables S3 and S4. Neither set is a certainty-of-evidence grade: both describe provenance only.

Three kinds of statement are kept separate throughout the text and the tables. Where a guideline, regulatory or product document is the source of a position, it is named and cited and its wording is not restated as though it were the authors’ own. Where a position rests on published study evidence, that evidence is given with the population in which it was generated. Where a position is the authors’ own synthesis, it is introduced as a proposal or a suggestion, and where no directly applicable data were identified it carries the [CV] label in Section 8 and Supplementary Tables S3 and S4. Where studies disagree the disagreement is presented rather than reconciled; where the literature is empty this is stated as an absence of evidence rather than filled by inference. Both conventions were applied by reading the cited source rather than by rating it, so that each label is fixed by the source to which it is attached and can be checked against it. The labels were assigned through direct assessment of the cited sources and were not generated through an independent duplicate-assignment procedure. Where classification required clarification, the underlying source was re-examined to determine the appropriate provenance label.

### 2.5. Transferability of Evidence from Non-Pregnant Populations

Where a position had to rest on evidence generated outside pregnancy, its transferability to pregnancy was judged qualitatively before it was applied, and the result is stated in the text where relevant and recorded by the [EX] label in Section 8 and Supplementary Tables S3 and S4.

Several considerations recurred. One was whether pregnancy alters the mechanism on which a finding depends, which is why the ambulatory venous hypertension model was not carried over to normal pregnancy (Section 3.3). Another was whether the gestational circulation changes the quantity being measured, as with reflux thresholds and diameter norms derived in non-pregnant subjects (Section 4.2). A third was whether the gravid uterus introduces an effect with no counterpart outside pregnancy, as with iliocaval compression and examination posture (Section 3.2 and Section 4.2). For drugs, a further consideration was whether placental transfer, altered pharmacokinetics or foetal safety make non-pregnant efficacy data insufficient on their own, which is why efficacy evidence for venoactive agents was not treated as grounds for use in pregnancy (Section 6.1). The last was whether a pregnancy-specific guideline or regulatory statement already addresses the question, in which case that statement governs. Extrapolation was accepted where the underlying mechanism is not plausibly altered by pregnancy and no pregnancy-specific evidence was identified; where transferability was doubtful and the clinical consequence material, the uncertainty is stated in the text rather than resolved.

### 2.6. Interpretation of Discordant Findings

Differences in population, pregnancy status, gestational age at measurement, parity, outcome definition, measurement technique, study design, sample size, duration of follow-up and clinical setting were considered in interpreting discordant findings, and are named in the text where they are identifiable. Where an explanation for a discordance is plausible but has not been demonstrated, it is given as a possibility and not as a finding.

Five disagreements are handled in this way. Calf pump function has been measured by two techniques with opposite results, so that method and sample are confounded (Section 3.2). Whether pregnancy produces new reflux is divided across three small serial studies with differing populations (Section 3.3). Whether parity independently determines varicose disease is divided between cohorts that adjust for age and family history and those that do not (Section 3.4). The reported time course of postpartum regression differs between the only primary cohort to measure it and a review restating a shorter interval (Section 7). And the wide spread of reported prevalence figures, within pregnancy and outside it, is treated throughout as a consequence of case definition rather than of biology (Section 1 and Section 4.1).

## 3. Venous Pathophysiology in Pregnancy

Section 3, Section 4, Section 5, Section 6 and Section 7 present the review’s substantive findings, beginning with the pathophysiology on which the diagnostic and management sections rest. This section concerns venous changes arising during pregnancy; it does not apply to women entering pregnancy with established chronic venous disease. The venous system of pregnancy is not passively overloaded but actively remodelled, regionally heterogeneous and, in most women, largely restored after delivery. Which changes are adaptive, which pathological and which reversible determines whether a third-trimester finding represents persistent disease or a physiological state. Figure 1 illustrates the principal mechanistic relationships, while Supplementary Table S1 provides their quantitative basis and source populations.

### 3.1. Hormonal Influences: Plausible Mechanism, Absent Human Confirmation

The hormonal hypothesis is the oldest and least directly demonstrated explanation, its support being receptor-level rather than physiological. Progesterone receptors were detected in 90% of varicose saphenous vein specimens from men and from pre- and postmenopausal women, none pregnant [34], establishing biological plausibility but not that gestational progesterone concentrations produce measurable venous relaxation. No identified study makes that link within the same subjects, and calf pump function did not correlate with progesterone concentrations in the one pregnancy study measuring both [35]. The oestrogen evidence is weaker and internally inconsistent: the same study found receptors absent or barely detectable in 25 of 30 specimens [34], whereas a separate immunohistochemical series reported them present in both sexes [36], with supporting associations that are menopausal rather than gestational [37] and experimental work that is animal [38]. Relaxin, frequently invoked, has substantial evidence for vasodilation, but exclusively arterial and renal [39]. No identified study measures vein wall matrix metalloproteinase activity, well characterised outside pregnancy [40], glycocalyx integrity, leukocyte–endothelial adhesion or endothelial activation in the lower limb of normally pregnant women.

Pregnant women demonstrate the physiological endpoint rather than its hormonal cause. Peripheral venous distensibility is greater in normal pregnancy than after delivery (*p* < 0.01), converging postpartum across normal and pre-eclamptic pregnancies [41] and consistent with earlier plethysmographic work [33]; that it is reduced in pre-eclampsia in proportion to severity [41] argues for an actively regulated rather than passively distended compartment. One small study found venous tone increased from the first trimester while lower-limb viscoelastic properties decreased in the third [42], which sits awkwardly with generalised progesterone-mediated relaxation. The defensible position is a regionally heterogeneous compartment in which central capacitance recruitment and dependent-limb dilation occur together rather than uniform hormonal venodilation.

### 3.2. Haemodynamic and Mechanical Loading

Plasma volume rises by approximately 6%, 18–29% and 42–48% across the trimesters, fastest in the first half of the second [24], and cardiac output peaks in the early third trimester at 1.5 L/min, or 31%, above non-pregnant values [25], so the lower limb receives a larger circulating volume through a more compliant bed at an unchanged hydrostatic column. Serial study of eight women showed venous filling index and junctional diameters rising to the late third trimester and returning postpartum; all filling-index values remained within the normal range, and no previously unaffected woman developed obstruction or varicose veins [43]. Flow velocity falls as diameter rises [44]: popliteal velocity and flow in late pregnancy were about one third lower than in non-pregnant women (both *p* < 0.0001), lower still in oedematous legs, the measured values being given as mean ± SEM in Table S1 [45]. This is the stasis limb of Virchow’s triad in numbers. Calf pump function has rarely been measured and the two studies disagree: strain-gauge plethysmography found it pathological in 17% of 24 normal pregnancies, with symptoms tracking the abnormality and virtually disappearing postpartum [35], whereas air plethysmography in healthy primigravidae found ejection fraction and residual volume fraction unchanged throughout [46]. A minority therefore develop measurable pump impairment, and it resolves.

Mechanical obstruction is the longest documented mechanism, venography having established that caval flow is substantially impeded supine at term [26], with haemodynamic effects varying by angle of lateral tilt [47]. Left-sided predominance is the clinically important refinement, usually attributed to left common iliac vein compression, which is present in many asymptomatic adults [48,49], so the substrate pre-exists pregnancy. Pregnancy-specific evidence is consistent: flow velocity was slower in the left than the right leg and increased in both in the left lateral position [44], while serial duplex found dilation maximal in the superficial system and more pronounced on the left [50]; the positional finding is the basis for advising left lateral rest in the second half of pregnancy. Saphenous dilation is detectable in early pregnancy [51], before the uterus is large enough to compress the iliocaval segment, so mechanical obstruction cannot be the initiating mechanism even if it dominates later.

### 3.3. Reflux, Reversibility and the Limits of the Venous Hypertension Model

Whether pregnancy causes reflux, unmasks it, or merely coexists with it is the central unresolved question, and the literature is genuinely divided. Two serial duplex studies found no new reflux in six women without venous disease followed monthly despite significant junctional and femoral dilation [43] and in 43 women scanned up to six weeks postpartum [27]. Against these, a prospective cohort of healthy primigravidae found reflux in 5% in the second trimester rising to 15% by the third, none postpartum [46]. Sample sizes are small in all three, and the discordance may reflect population as much as method.

The most defensible synthesis is that pregnancy produces new reflux in a minority of previously competent limbs, that it is usually transient, and that the dominant change is dilation rather than valvular failure. Reflux on a third-trimester duplex should not therefore be assumed to represent established permanent disease. And the absence of demonstrable venous hypertension in one pregnancy study that assessed it [46], with normal ambulatory venous pressures, estimated by air plethysmography, in another [43], means the ambulatory venous hypertension model underpinning conventional CVD pathophysiology [52] should not be transferred wholesale to normal pregnancy.

Reversibility is similarly contested, and the disagreement maps onto which vein is measured. Deep and junctional dilation reverses essentially completely [43,46]. The largest superficial-vein study agreed: in 66 women with varicose veins (85 affected extremities), great and small saphenous and largest-varicosity diameters increased between the first and third trimesters and decreased again postpartum (*p* < 0.001 for both), postpartum values not differing significantly from first-trimester values [53]. A smaller study measuring erect long saphenous cross-sectional area concluded the opposite, finding postpartum areas still larger than in a separate nulliparous comparison group [51]; comparing different women rather than the same women before conception weakens that inference, though an incompletely reversible superficial residue remains the most plausible structural explanation for a cumulative parity effect. Oedema formation is separately multifactorial [54], so its presence carries little diagnostic information about the venous system. The principal pregnancy-associated venous changes, their peak timing and their postpartum reversibility are summarised in Table 1.

### 3.4. The Pre-Existing Substrate

Pregnancy acts on a substrate substantially determined before conception, monogenic and polygenic evidence both indicating that susceptibility to reflux is largely pre-set [55,56]. Family history is consistently the strongest clinical risk factor in pregnancy cohorts, carrying a relative risk of 5.8 (95% CI 3.8–8.9) for disease arising during pregnancy against 2.0 (95% CI 1.3–2.9) for multiparity versus primiparity [57] and surviving multivariable analysis alongside maternal age in a second cohort [5]. Duplex-detected reflux at booking in 329 UK women, however, showed no such relationship [58].

Parity is frequently cited as a risk factor, but the supporting evidence is inconsistent. Supporting it are the pooled odds ratio above [7], a dose–response of 1.14 per birth [59] and a per-pregnancy hazard increase in 156,622 women [60]. Opposing it, the Edinburgh Vein Study found no association between previous pregnancy and duplex-detected reflux (adjusted odds ratio 0.96, 95% CI 0.71–1.29) [61], and over 13 years identified neither parity among incident risk factors nor any sex difference in varicose vein incidence [62]. Two observations may account for much of this, although neither has been shown to do so: parity correlates with age and family history, so models adjusting for both may over-control; and parity appears to influence varicose disease without altering saphenous anatomy, great saphenous reflux prevalence not differing across women with 0 to ≥4 pregnancies [63]. Both would follow if pregnancy-associated varicose disease were disproportionately of non-saphenous, pelvic-escape origin, a possibility supported by the excess of dilated incompetent ovarian veins in parous compared with nulliparous asymptomatic women [64]. Other risk factors also behave differently: obesity robustly predicts incident chronic venous insufficiency in the general population [62], yet two pregnancy studies found no association with pregnancy-related varicose veins [57,58], so effect sizes derived from non-pregnant adults should be applied with explicit caution.

## 4. Clinical Presentation and Diagnostic Pitfalls

### 4.1. Classification and Its Limits in Pregnancy

Under the VEIN-TERM consensus, chronic venous insufficiency is reserved for advanced disease producing oedema, skin changes or ulceration, CEAP classes C3–C6 [6,65]. Applying that term to the whole spectrum, as much of the pregnancy literature does, converts telangiectasia in a young primigravida into a diagnosis implying skin change and ulcer risk. CEAP itself applies with three caveats. It has no temporality or reversibility qualifier, so a woman classified C3 at 34 weeks and C0–C1 six months later cannot be recorded as transient. Its secondary-extravenous aetiological subclass names obesity and extrinsic venous compression but not the gravid uterus, the archetypal reversible extrinsic compression [66]. The vulvar, perineal and pelvic-origin varices most typical of pregnancy fall outside the leg-oriented C classes, requiring the separate Symptoms–Varices–Pathophysiology instrument alongside it [67]. We therefore suggest that a class assigned during pregnancy carry its gestational age and be recorded as provisional; this is a reporting convention proposed here, not a CEAP rule. Neither the Venous Clinical Severity Score [68] nor any venous-specific patient-reported outcome measure [69,70] has been validated in pregnancy, and severity items covering oedema and pain cannot separate venous from gestational causes, so serial scores are likely to inflate systematically.

Varicose veins define the clinical problem. New varicose disease was reported by 132 of 474 previously unaffected women (28%) in one postpartum survey [57], while at booking superficial venous reflux was already detectable on duplex in 83 of 329 women (25%) [58], so much of what is later attributed to pregnancy predates it or arises very early. Against a background annual incidence in general adults of 1.4% (95% CI 1.1–1.7) [62], an incidence of the order of 25–30% over nine months is nonetheless a genuine excess. Oedema is hardest to assign, physiological dependent and venous oedema being clinically indistinguishable with no large trimester-resolved prevalence study available [12], while skin changes and ulceration are largely confined to women entering pregnancy with pre-existing advanced disease, usually post-thrombotic. Any pregnancy framework is therefore in practice one for C0–C3 disease. The disease is symptomatic rather than cosmetic, symptoms increasing through pregnancy and virtually disappearing postpartum as calf pump function normalises [35,71].

### 4.2. Why Diagnosis Is Difficult

Three problems converge. Symptomatic overlap is near-complete, since aching, heaviness, swelling, nocturnal cramps and restlessness arise from normal pregnancy, from chronic venous disease and from non-venous conditions; restless legs syndrome alone affects roughly one in four to one in five women in the third trimester (pooled prevalence 22.9%, 95% CI 14.7–33.8%) [11]. The reference standards were built outside pregnancy: duplex thresholds for pathological reflux, greater than 0.5 s in superficial and calf veins and greater than 1.0 s in femoral and popliteal veins, derive from a non-pregnant cohort [72]. The 0.5 s criterion is used in international consensus [73], and both thresholds are reproduced in current guidance [74], yet have never been re-derived in a venous system carrying 40% more circulating volume with dilated veins and reduced flow velocity. And the condition appears substantially under-recorded: the coded prevalence of lower-extremity venous insufficiency in 256,531 vaginal births was 1.13% [4], against a duplex-ascertained prevalence of 25% at antenatal booking in a differently ascertained population [58]. The two figures are not directly comparable, differing in country, case ascertainment, sampling frame, timing of assessment and probably phenotype definition, so the gap cannot be read as an epidemiological difference. Its magnitude nonetheless points to under-recognition or under-documentation rather than to any measured deficit in imaging use.

Examination identifies vulvar, perineal and gluteal varices that are missed if not sought, but it cannot exclude deep vein thrombosis, and the specificity problem is structural: two of the three components of the LEFt rule, left leg symptoms and a calf circumference difference of at least 2 cm, are exactly what gestational venous distension and dependent oedema produce [15]. Examination should be standing where tolerated, since reflux is under-detected supine [73]; in advanced pregnancy this is often uncomfortable and prolonged supine positioning is inadvisable because of aortocaval compression [26,47]. Reverse Trendelenburg positioning is the accepted alternative when standing is not possible [74], and we suggest adding left lateral tilt in late pregnancy for the same reason.

Duplex ultrasonography is the test of choice for lower-limb venous reflux [75] and the principal modality for routine venous assessment in pregnancy; other modalities are required for specific suspected complications, as set out below. A complete assessment is an anatomical and haemodynamic map covering junctional competence, truncal and tributary reflux, perforators, deep system patency and, where indicated, pelvic escape points—not a two-point compression study for thrombus exclusion [73,76]. Its safety is frequently misunderstood, causing harm through unnecessary deferral: restrictive early-pregnancy statements on spectral Doppler are scoped to insonation of the embryo or foetus, AIUM stating that scanning the maternal uterine arteries in the first trimester is unlikely to have foetal safety implications provided the embryo or foetus lies outside the Doppler beam [77]. No statement identified here restricts maternal lower-limb venous duplex, which may be performed whenever clinically indicated at any gestational age. Interpretation nonetheless needs caution, since venous diameters increase substantially and reversibly through gestation, the common femoral vein by approximately 22% and the saphenofemoral junction by approximately 48% between the first and late third trimesters [43], with superficial diameters returning to first-trimester values postpartum [53]. Reflux durations in later pregnancy may therefore exceed conventional thresholds in women whose venous system will be entirely normal six months later. We propose three rules. Record gestational age on every report; describe findings rather than assign definitive disease labels; and do not commit a woman to a permanent CEAP class, or to a treatment plan, on a third-trimester study alone.

### 4.3. Recurring Pitfalls and Red Flags

Several errors recur; Table S2 sets out the supporting data and the assessment indicated for each. Four concern under-investigation. Unilateral leg swelling should not be dismissed as physiological, since physiological oedema is bilateral and dependent whereas pregnancy-associated deep vein thrombosis is left-sided or bilateral in 82.2% (95% CI 75.1–87.5) of cases [14]. An incomplete leg study can miss the majority pattern, most thromboses in pregnancy being proximal, sparing the calf and involving the iliac or femoral veins [13]. The ASH panel accordingly suggests serial compression ultrasound or magnetic resonance venography after a negative initial study that has imaged the iliac veins [78], a single complete whole-leg study having left a three-month symptomatic venous thromboembolism rate of 1.1% (95% CI 0.3–4.0%) [79]. D-dimer interpreted outside a validated algorithm is uninformative, since by the third trimester essentially no healthy woman remains below the conventional 0.50 mg/L threshold [80], and the 95th centile exceeds the non-pregnant cut-off at every gestational time point studied [81]. Guidance is divided between RCOG, which advises against its use in pregnancy [82], and the 2019 ESC guideline, which gives a Class IIa recommendation that it be considered alongside clinical prediction rules [83]. The SOMANZ position statement does not currently recommend the addition of D-dimer testing once pulmonary embolism is considered the most likely diagnosis [84]. Prospective management studies reconcile the positions: D-dimer is usable within a defined pathway at algorithm-specific thresholds, not as a standalone test [29,85]. Nor does clinical probability exclude thrombosis—external validation of the LEFt rule found sensitivity and negative predictive value of 100% but specificity of only 32%, with an upper confidence bound for thrombosis of 7.9% among women with no criteria present [16].

Three further errors run the other way. Over-reading pregnancy-related venous dilation commits a young woman to a diagnosis she may not have, since increased diameters and reflux durations marginally beyond threshold are expected in late pregnancy and reverse after delivery [43,46,53]. Over-diagnosing iliac vein compression is analogous: radiological left common iliac vein compression is frequent in asymptomatic non-pregnant adults [48,49], so demonstrating it in a pregnant woman with a swollen left leg establishes neither causation nor an indication for intervention. Failing to reassess after delivery is the third, since most gestational venous change reverses [46,53] and a minority does not [51]. A final error inverts all of them: deferring necessary imaging out of misplaced concern for foetal safety. Lower-limb venous duplex involves no foetal insonation, the concern that restrictive Doppler statements address [77], and where thoracic imaging is indicated, estimated foetal doses are low for both ventilation–perfusion scintigraphy and CT pulmonary angiography [86].

**Figure 2:**
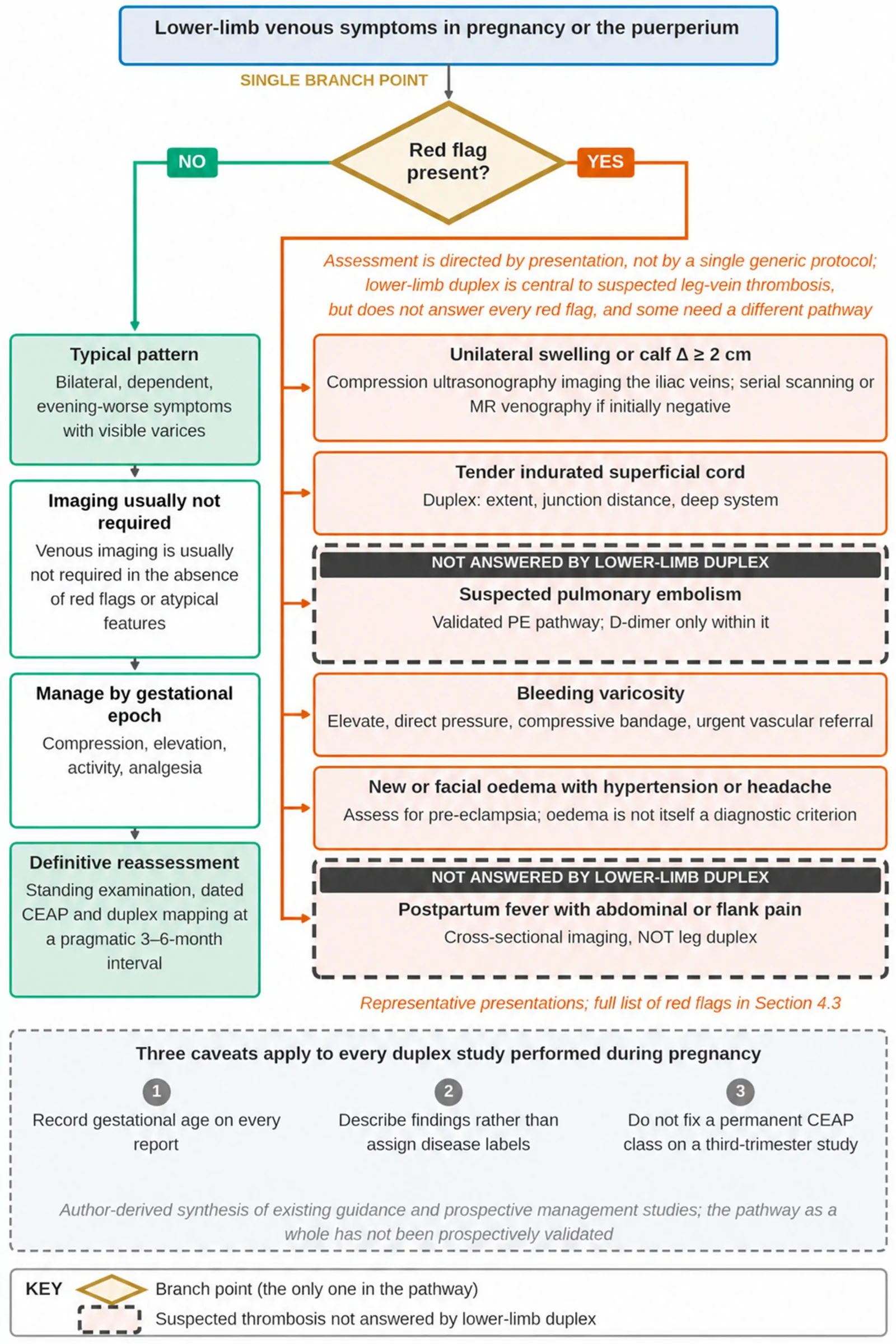
Diagnostic pathway for lower-limb venous symptoms in pregnancy and the puerperium. The single branch point is the presence or absence of a red flag. Where none is present, venous imaging is usually not required for a typical bilateral dependent pattern, and management proceeds by gestational epoch, with reassessment at a pragmatic 3–6-month postpartum interval. Where a red flag is present, assessment is directed by presentation: the presentations shown require different investigations and are representative rather than exhaustive, the full list of red flags being given in Section 4.3. Lower-limb duplex is central to suspected leg-vein thrombosis but does not answer every red flag, and some red flags require a different pathway; suspected pulmonary embolism, which requires a validated pulmonary embolism pathway, and postpartum fever with abdominal or flank pain, which requires cross-sectional imaging, carry a heavy dashed border and an explicit label defined in the in-figure key. The dashed panel carries the three interpretive caveats applying to every duplex study performed during pregnancy. The pathway is an author-derived synthesis assembled from existing guidance and prospective management studies; it has not itself been prospectively validated. The red-flag branches follow the cited sources; the no-red-flag branch, including the pragmatic 3–6-month reassessment interval, and the three caveats are author-derived. Supporting values and sources are given in Table S2 and, for bleeding from a varicosity, in Section 4.3. CEAP, Clinical–Etiological–Anatomical–Pathophysiological classification; MR, magnetic resonance; PE, pulmonary embolism; Δ, difference in calf circumference between limbs.

The differential is broad and mostly non-venous. Pre-eclampsia must be considered whenever oedema is new, rapid or facial, although peripheral oedema is not itself a diagnostic criterion [87,88]; lymphoedema, lipoedema, drug-induced oedema and cardiac, renal or hepatic causes deserve consideration when distribution or time course is atypical. The following warrant urgent evaluation rather than reassurance: new unilateral leg swelling, particularly left-sided, or a calf circumference difference of at least 2 cm [13,14,15]; acute severe leg or buttock pain with swelling, suggesting iliofemoral thrombosis [13]; a tender, indurated, erythematous cord along a superficial vein, requiring duplex assessment of extent and of the deep system [28,89]; and symptoms suggesting pulmonary embolism, requiring a validated pathway [29,83,85]. Three further presentations require the same urgency: bleeding from a varicosity, requiring immediate elevation, direct compression and urgent vascular referral [9,90]; rapidly progressive skin change or ulceration over a varicosity; and postpartum fever with abdominal or flank pain, raising the possibility of ovarian vein thrombosis or septic pelvic thrombophlebitis, diagnosed on cross-sectional imaging rather than leg duplex [91,92]. Figure 2 sets out the resulting pathway.

## 5. Conservative Management During Pregnancy

Treatment goals in pregnancy differ from those outside it. Where guidelines recommend intervention over long-term compression for symptomatic truncal reflux in non-pregnant women [6,75], intervention is unavailable to most pregnant women [9], and the realistic objectives are to relieve symptoms, limit oedema, maintain mobility, detect complications, explain the likely course accurately and arrange postpartum reassessment. We suggest three principles. Distinguish physiological change from disease and say which you think it is: telling a woman that her veins are enlarged by pregnancy and will most likely improve after delivery is accurate and therapeutic, and is the substance of the only NICE recommendation on information-giving here [9]. Treat symptoms rather than images, since no pregnancy study shows that treating a duplex finding alters any clinical outcome. And keep chronic venous disease and venous thromboembolism separate while managing their overlap: gross or symptomatic varicose veins are a scoreable item in obstetric venous thromboembolism risk assessment [93] and superficial vein thrombosis markedly raises thromboembolic risk [28], but managing varicose veins does not itself make the thromboprophylaxis decision, which follows obstetric assessment [78,93].

### 5.1. Compression Therapy

Compression is the only chronic venous disease treatment recommended in pregnancy by more than one guideline body, and the one whose benefits are most often overstated. Four randomised or quasi-randomised pregnancy studies exist, and their results diverge informatively. The only trial designed to test prevention was negative: compression did not prevent new varicose veins (emergent varicosities in 7 of 14 controls versus 5 of 12 and 8 of 14 in treated groups, *p* = 0.94), although it reduced emergent third-trimester reflux at the saphenofemoral junction (1 of 27 versus 4 of 15 women, *p* = 0.047) and improved leg symptoms (*p* = 0.045) [94]. Two later, probably overlapping, trials from one group reported much larger benefits, including reductions in great saphenous diameter, CEAP progression and symptom prevalence [71] and near-abolition of the gestational increase in calf and ankle circumference [95]. Given the small samples, incomplete blinding and reliance on subjective or surrogate outcomes, these effects are best read as directional rather than as precise estimates. A non-randomised study found that one week of below-knee compression raised popliteal venous velocity by 43.5% and flow by 47.6% in oedematous legs (both *p* < 0.0001) [45], although the inference that deep vein thrombosis could thereby be prevented rests on surrogate endpoints, and no pregnancy trial has reported a clinical thromboembolic endpoint. The Cochrane synthesis, its search predating three of these studies, found no clear difference in lower leg volume between compression and rest in a single 35-woman trial [8].

Compression has therefore not been shown to prevent varicose veins in pregnancy; the NICE evidence review, which graded the relevant outcomes as low quality because of inadequate allocation concealment and blinding, reached the same conclusion [94,96], and a German expert review likewise states that no prophylactic effect has been demonstrated [31]. The apparent conflict with the report of prevented CEAP progression [71] probably reflects different measures—new visible varicosities versus class change where venous signs already existed. The Cochrane review of stockings as initial treatment of varicose veins without ulceration found subjective symptom improvement in all four studies reporting it, certainty low to very low, and concluded that evidence is insufficient to determine whether stockings are effective as sole and initial treatment [97]. Compression is thus recommended on physiological rationale, safety, low cost and consistent symptomatic benefit in small trials, rather than on high-certainty evidence of disease modification, and is best presented to women on that basis.

No dose-finding study exists in pregnancy. In the non-pregnant dose–response literature, 10–21 mmHg suffices for symptom relief in CEAP C0–C1 and 15–21 mmHg helps prevent oedema [98]. Pregnancy goals sit at that lower end, and three considerations reinforce a low-pressure default: class I did not differ significantly from class II in outcome in an intra-individual comparison [94]; higher classes are associated with poorer tolerance and greater application difficulty [99]; and donning becomes harder as the abdomen enlarges. We consider knee-length garments usually preferable, and no pregnancy evidence favours thigh-length. The anti-embolism stockings recommended by RCOG at 14–15 mmHg for hospitalised women with a contraindication to low-molecular-weight heparin [93] are a different device, pressure and indication; the two studies should not be merged.

Adherence deserves as much attention as pressure. The principal adherence literature formally excludes pregnancy, a scoping review of 69 articles having excluded time-limited stocking wear while reporting adherence from 20.3% to 95% [99], and a systematic review identifying five recurring barrier themes—physical limitations, health literacy, discomfort, financial issues and psychosocial issues—each intensified by pregnancy [100]. Pregnancy-specific tolerability data include those from the 20–30 mmHg trial, in which 36.67% reported difficulty wearing the garments, yet all reported a difference in leg symptoms [95]. We suggest prescribing a garment the woman can put on, anticipating intermittent rather than continuous wear, and treating partial adherence as a normal outcome. NICE advises against compression hosiery to treat varicose veins unless interventional treatment is unsuitable, but recommends considering it for symptom relief of leg swelling associated with varicose veins during pregnancy [9]; the European guideline addresses pregnant women within its chapter on special patient characteristics [6]; and the most widely cited international compression consensus mentions pregnancy only in a single ungraded statement [101]. Compression is recommended in pregnancy precisely because intervention is unavailable, not because it works better in pregnant women.

### 5.2. Activity, Position and Adjunctive Measures

Measures beyond compression have modest and mostly indirect evidence. Guidance recommends at least 150 min of moderate-intensity aerobic activity weekly on general health grounds [102]; the venous benefit is inferred from calf pump physiology [103] rather than demonstrated. Leg elevation is paired with graduated compression for acute deep vein thrombosis in national guidance [82] and served as the comparator in a trial of water immersion [8]. Upright water immersion reduced leg volume in nine women with marked dependent oedema without haemodynamic compromise [104], and modified Buerger–Allen exercises in 105 women with late-pregnancy oedema reduced circumference, volume and pain (*p* < 0.05) [105]. These measures rest on small, mostly unblinded studies and are best regarded as reasonable adjuncts rather than established venous therapies. Avoiding prolonged static standing is advised on mechanistic grounds [3,59,103] but has not been tested in pregnancy. Excessive gestational weight gain was among the factors associated with subsequent varicose veins in a large multicentre health-system cohort [60], though the relationship with body mass index is inconsistent [57,58]; gestational weight management belongs in antenatal care on its own merits, without implying that a woman’s venous symptoms are due to her weight.

## 6. Pharmacological and Interventional Management

Table S3 summarises the options, their evidence basis and their appropriate timing.

### 6.1. Venoactive Drugs and Analgesia

In general adults, the Cochrane phlebotonics review found that these agents probably reduce oedema slightly (risk ratio 0.70, 95% CI 0.63–0.78; moderate certainty), with no clinically meaningful improvement in quality of life and an excess of adverse events [106]. Agent-specific syntheses report larger benefits [107], and the European guideline recommends that they be considered in symptomatic patients not undergoing intervention, Class IIa with Level A evidence [6]. None of that evidence includes pregnant women, and the recommendation does not address pregnancy.

Pregnancy-specific efficacy data exist for one agent. Rutosides reduced varicose vein symptoms versus placebo in a single trial of 69 women (risk ratio 1.89, 95% CI 1.11–3.22; moderate-quality evidence), with no significant difference in venous complications or side effects [8]; that trial enrolled women at 28 weeks and treated for eight weeks [31]. Safety data, not efficacy, are the limiting factor. The oxerutin leaflet states that use is not recommended in the first trimester as a general safety precaution [22], and European Union herbal monographs for horse chestnut seed, *Ruscus aculeatus* rhizome and red vine leaf each state that safety during pregnancy and lactation has not been established and that use is not recommended in the absence of sufficient data [19,20,21]. The monograph formulation records an absence of study rather than observed harm, and should not be reported as a contraindication; equally, it cannot be read as reassurance. A German expert review concludes that phlebotonics should be avoided in pregnancy and lactation because of sparse data, despite quoting the positive rutoside trial [31].

Routine initiation of venoactive drugs during pregnancy therefore cannot be recommended, because pregnancy-specific safety data are insufficient; the historical rutoside efficacy signal rests on a single small trial reported decades ago, and efficacy evidence does not substitute for safety evidence. Where an individual licenced product is nonetheless considered, we suggest that the decision rest on that product’s own current information rather than on the class, follow national licencing, which differs materially between countries, and be documented as a shared decision. We also suggest direct enquiry about over-the-counter venoactive preparations, which are widely available and often taken without disclosure.

Simple analgesia is often what a woman with symptomatic varicosities actually needs, and the options narrow sharply after 20 weeks. The FDA advises that pregnant women should not use non-steroidal anti-inflammatory drugs at 20 weeks or later unless specifically advised by a health care professional, because of foetal renal dysfunction causing oligohydramnios and sometimes neonatal renal impairment, with the established risk of premature ductal closure applying from approximately 30 weeks [23]. The advice covers all prescription and over-the-counter NSAIDs, the products listed including topical diclofenac formulations, with the exception of NSAIDs administered directly to the eye and of low-dose aspirin used for pregnancy-related conditions under medical direction [23]. Paracetamol remains first-line, a national position statement recommending it when medically indicated at recommended doses for the shortest duration required [108]. From mid-pregnancy, compression, elevation, activity modification and paracetamol constitute the entire symptomatic armamentarium for uncomplicated chronic venous disease.

### 6.2. The Boundary with Thrombosis

Anticoagulation has no role in uncomplicated chronic venous disease and becomes relevant only with thrombotic complications. Low-molecular-weight heparins are the agents of choice, national guidance stating that there is evidence they do not cross the placenta and that they are safe in breastfeeding; vitamin K antagonists should not be used antenatally because of embryopathy, although warfarin is compatible with breastfeeding [82,93], and direct oral anticoagulants should be avoided in pregnancy and are not recommended in breastfeeding [83,93].

Superficial vein thrombosis converts a chronic venous problem into an acute one, and its management in pregnancy is almost entirely extrapolated. Of 211 women with antepartum superficial vein thrombosis, 22 (10.4%) later developed venous thromboembolism against 25 (0.1%) of matched controls, a hazard ratio of 83.3 (95% CI 46.3–149.7) [28]. Even allowing for surveillance bias, one in ten justifies taking the diagnosis seriously. Duplex ultrasonography should be performed to define thrombus extent, proximity to the deep system and any concomitant deep vein thrombosis: concurrent deep vein thrombosis or pulmonary embolism is present at diagnosis in approximately a quarter of general-population patients with symptomatic superficial vein thrombosis [89]. The ASH panel suggests low-molecular-weight heparin over no anticoagulant, a conditional recommendation on low-certainty evidence, with no agreement on dosing but general agreement among panel members on treatment for the remainder of pregnancy and 6 weeks postpartum [78]. A regional position paper proposes consensus-based doses and durations tiered by thrombus location, with junction-distance thresholds of 10 cm and 5 cm [30]. The pivotal randomised trial excluded thrombosis within 3 cm of the saphenofemoral junction [109], no pregnancy-specific junction-distance threshold has been validated, and the largest pregnancy dataset recorded no anatomical extent data [28]. NSAIDs, usual for this indication outside pregnancy, are to be avoided from 20 weeks unless specifically advised [23]. Thromboprophylaxis decisions follow obstetric rather than venous assessment, but gross varicose veins—symptomatic, above knee, or associated with phlebitis, oedema or skin changes—are a scoreable risk factor in the RCOG antenatal and postnatal risk assessment [93], so a vascular assessment documenting this phenotype should reach the obstetric team.

### 6.3. Interventional Treatment

Four categories are routinely conflated: formal contraindication in a named authoritative document; non-recommendation by a named guideline; deferral by convention; and insufficient evidence. The clearest statement is a negative recommendation rather than a contraindication—NICE states that interventional treatment for varicose veins should not be carried out during pregnancy other than in exceptional circumstances, the worked example being bleeding varicosities [9,96]. German guidance goes further, listing known pregnancy among the absolute contraindications to elective open varicose vein operations [17], while the International Union of Phlebology sclerotherapy consensus places pregnancy, the postpartum period and breastfeeding among the relative contraindications [18].

Regulatory positions diverge instructively. The United States polidocanol microfoam label does not list pregnancy as a contraindication, noting that published case reports have not identified drug-associated risk while adding that there is minimal benefit in treating lower extremity varicosities during pregnancy because they may spontaneously regress postpartum [110]: the argument there is futility rather than demonstrated harm. In Europe the position is more restrictive—polidocanol crosses the placenta, animal studies showed embryo- and fetotoxic effects at maternally toxic doses, no human teratogenicity or breast-milk data exist, and both the German sclerotherapy guideline and the manufacturer’s product information list pregnancy as a contraindication, as reported in a German expert review [31]. Published case material on inadvertent sclerotherapy in unrecognised pregnancy reports no increased risk at conventional doses [111], but absence of reported harm in small case series is not demonstrated safety.

For every other modality the position is more accurately described as deferral by convention or absence of evidence. Pregnancy was a formal exclusion criterion in the major randomised trials of endovenous treatment, and breastfeeding in one of them [112,113], and no guideline identified here addresses thermal ablation, mechanochemical ablation or cyanoacrylate closure in pregnancy specifically. Tumescent anaesthesia is a particular gap, using total lidocaine doses far above labelled maxima and never having been studied in pregnancy—an absence of data rather than a safety signal.

Two arguments support deferral beyond guideline prohibition. The first is spontaneous regression: superficial vein diameters return to first-trimester values postpartum [53] and reflux acquired in pregnancy resolves in healthy primigravidae [46], so treating a vein that will normalise is unnecessary. The second is durability—prior parity independently predicted saphenofemoral reflux recurrence after ligation and stripping (odds ratio 2.69, 95% CI 1.45–4.97) [96,114], and five of eight women who became pregnant after coil embolisation for pelvic vein reflux had recurrent truncal pelvic reflux [115]. Recurrence is common regardless of pregnancy, reaching 62% at 11 years [116], so pregnancy is one contributor among several rather than a reason to withhold treatment from a symptomatic woman between pregnancies.

Deferring treatment is not deferring assessment. German guidance explicitly requires referral to a vein specialist for any patient with varices in pregnancy, variceal bleeding, superficial vein thrombosis or a venous ulcer [17], and NICE requires immediate referral of bleeding varicose veins to a vascular service [9]. Bleeding from a varicosity is the principal recognised exception: rupture can produce significant, occasionally fatal haemorrhage, and immediate management is elevation, direct pressure then a compressive bandage, resuscitation if blood loss is large, and urgent referral [90], with multi-society consensus advising direct compression and sclerotherapy before suture ligation [117]. None of these documents addresses pregnancy: the technique is established, its application in pregnancy is not.

## 7. Vulvar and Pelvic Venous Disease

Vulvar and perineal varices are the phenotype most characteristic of pregnancy and the most often overlooked, being rarely volunteered and rarely sought. Prevalence has been estimated at 18–22% of pregnant women [118] and at 8–22% in a later review, rising above 20% with concurrent lower-limb varices [119]; no denominator-based screening study exists, so the spread reflects ascertainment as much as biology. They characteristically appear at 18–24 weeks in a first pregnancy and 12–18 weeks in subsequent ones [118].

Management in pregnancy is conservative, with supportive garments and with surgery reserved for complications [118,119]. Thrombophlebitis occurred in 2 of 40 pregnant women in one series, at 28 and 32 weeks [118]. Bleeding at delivery is described as extremely rare and readily controlled by ligation, but no study gives a rate and the whole published experience of severe cases consists of case reports [118]. That missing denominator is itself the argument against prophylactic caesarean section: a systematic review located only 11 published cases of very large vulvovaginal varicosities, of whom 6 delivered by caesarean section, 4 of those without a mandatory obstetric indication [120]. On the available evidence, we suggest that mode of delivery be decided on obstetric grounds with vascular input rather than on the appearance of the varices alone.

Natural history is better documented for vulvar varices than for any other pregnancy-related venous phenotype. Dilated vulvar veins persisted in only 20% of 25 women followed to at least one year; in most of the remainder they disappeared completely within 2–8 months (mean 5.8 ± 1.04 months, the dispersion measure not defined in the source), faster with shorter breastfeeding [118]. The widely repeated claim of resolution within six weeks postpartum [119] is unsupported by the only primary cohort measuring the time course; the discrepancy should be acknowledged rather than averaged. Varices still symptomatic past that interval can be treated by sclerotherapy or phlebectomy once the woman is no longer pregnant [117,118], though not without risk, a large vulvar haematoma requiring surgical drainage having been reported after phlebectomy with ovarian vein embolisation [121].

Pelvic-origin leg varices raise a parallel problem of interpretation. The Symptoms–Varices–Pathophysiology classification, the current framework for pelvic venous disorders, is not validated in pregnancy and offers no guidance on the pregnant patient [67]. Non-saphenous reflux affects roughly one in five women presenting with varicose veins, and pelvic vein reflux around one in six [122]. Yet dilated, incompetent ovarian veins are common in asymptomatic women: 15 of 24 parous versus 1 of 10 nulliparous women (63% versus 10%, *p* < 0.05) in one computed tomography series [64]. Both observations must be held together: atypically distributed leg varices in a parous woman may warrant assessment above the groin, while a dilated ovarian vein is expected rather than diagnostic in itself. No pregnancy-specific data exist on the prevalence or course of perineal, inguinal, gluteal, obturator or sciatic escape points, and no pregnancy-validated imaging pathway exists. Where symptoms permit, definitive pelvic venous intervention is commonly deferred until after pregnancy, and the decision should be individualised in women planning further pregnancies, since truncal pelvic reflux recurred in five of eight women who conceived after coil embolisation, although no coil displacement occurred and the authors considered pregnancy after embolisation to be safe [115].

## 8. A Trimester-Stratified Clinical Management Framework

### 8.1. Status, Rationale and Limits

The framework presented below is an author-derived, evidence-informed and hypothesis-generating synthesis for the assessment and management of chronic venous disease across gestation. It is not a clinical prediction tool, a clinical guideline or a formal consensus recommendation, and it has not been prospectively validated or tested against clinical outcomes. Most of the positions it arranges are carried by the sources cited in Section 3, Section 4, Section 5, Section 6 and Section 7, distinguished there as pregnancy-specific evidence, extrapolated evidence or convention. Its principal contribution is their arrangement along the gestational axis on which the underlying decisions actually change. A limited number of elements, such as the pragmatic postpartum reassessment interval of Section 8.3, go beyond the cited sources; these author-derived elements are introduced as proposals or suggestions or labelled [CV]. Table 2 gives the framework at a glance and Table S4 presents it in full, epoch by epoch, with statements labelled there as pregnancy-specific [PS], extrapolated [EX] or convention-based [CV].

**Figure 3:**
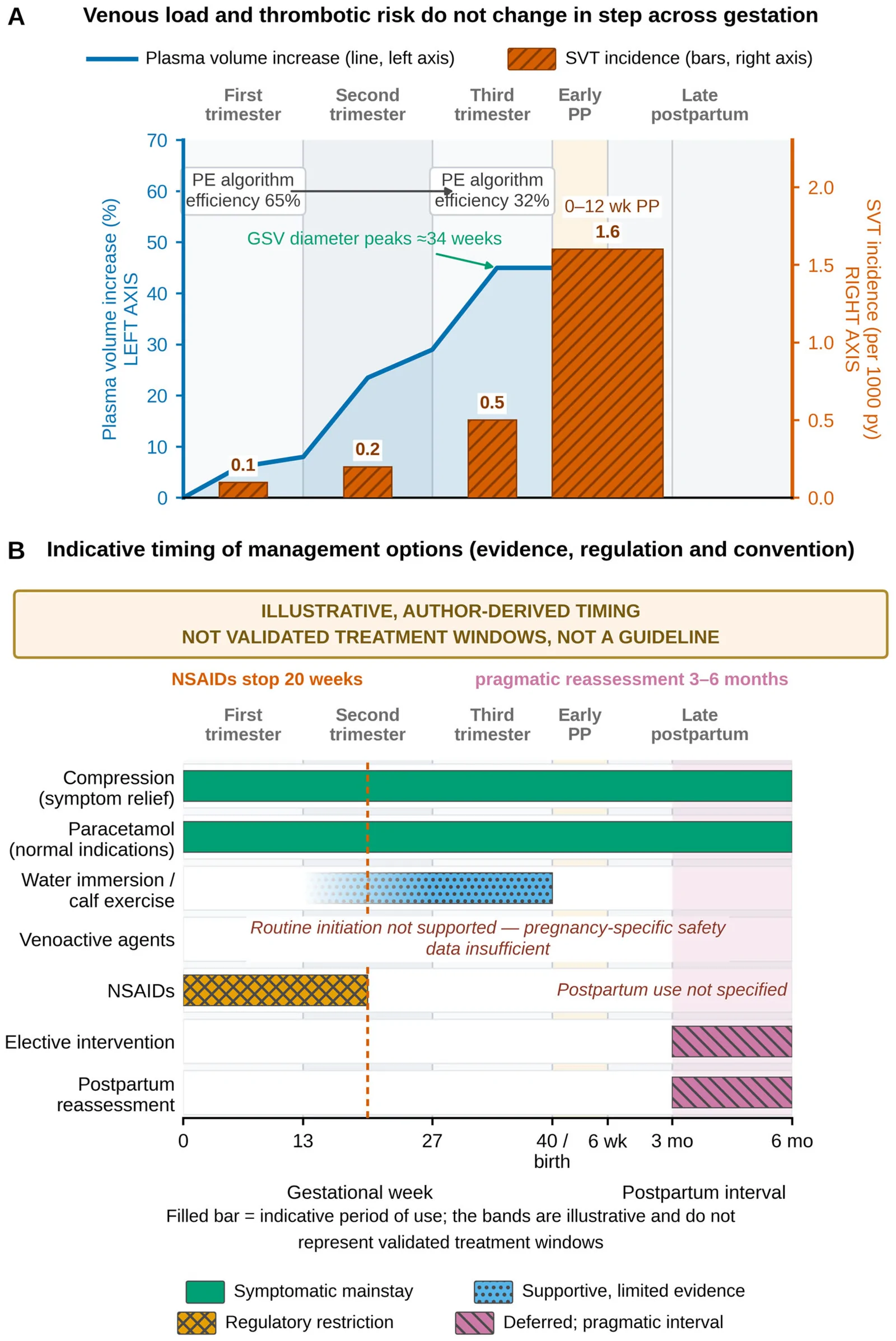
Gestational trajectory of venous load and thrombotic risk, with indicative timing of management options. The framework that timing panel (**B**) displays is author-derived, evidence-informed and hypothesis-generating: it was not produced by a formal consensus process, has not been prospectively or externally validated or tested against clinical outcomes, and is not a clinical guideline or consensus recommendation. (**A**) Plasma volume above the non-pregnant baseline (line, left axis) and superficial vein thrombosis incidence per 1000 person-years (bars, right axis), the two axes being distinguished by colour, an axis title naming the side, and a key within the panel, across the three trimesters and the postpartum period; the efficiency of a validated pulmonary embolism algorithm falls from 65% in the first trimester to 32% in the third [29]. The plasma volume line is limited to pregnancy, the period covered by the cited meta-analysis [24]. The postpartum bar gives the incidence over the first 12 postpartum weeks, the interval analysed in the source study [28]. (**B**) Indicative timing of management options based on available evidence, regulatory constraints and clinical convention. A filled bar denotes the period in which an option may reasonably be used; bar colour distinguishes symptomatic mainstay, supportive measure with limited evidence, regulatory restriction and deferral; the four categories are additionally distinguished by fill pattern, plain, dotted, cross-hatched and diagonal, so that they remain separable without colour. The timing bands are illustrative and do not represent prospectively validated treatment windows. Venoactive agents carry no bar because routine initiation during pregnancy is not supported by pregnancy-specific safety data. Compression is shown as a symptomatic measure and is not implied to modify disease. The dashed vertical marks the regulatory restriction on non-steroidal anti-inflammatory drugs from 20 weeks, and the shaded band the pragmatic, author-derived 3–6-month postpartum reassessment interval, which is not a validated biological threshold. The NSAID restriction applies to pregnancy [23]; the figure does not specify postpartum use. The graded start of the water-immersion and calf-exercise bar indicates that no starting gestation is defined. Values and their sources are given in Tables S1, S3 and S4. GSV, great saphenous vein; NSAID, non-steroidal anti-inflammatory drug; PE, pulmonary embolism; PP, postpartum; py, person-years; SVT, superficial vein thrombosis.

The rationale for a temporal axis is physiological rather than administrative: trimesters are not biologically discrete, but the clinically relevant variables change monotonically across gestation and no existing guidance organises management along that axis. Four decisions move with gestational age. The pharmacological repertoire narrows at 20 weeks with the NSAID restriction [23]; venoactive agents are not routinely initiated at any stage, and product labelling adds an explicit first-trimester restriction for the one agent with pregnancy efficacy data [22]. The imaging threshold falls as superficial vein thrombosis incidence [28] and thromboembolic risk rise while diagnostic algorithms lose efficiency [29]. Positional advice matters more once aortocaval compression becomes significant [44,47]. And examination technique must change in the third trimester [26,47]. Two decisions do not move: deferral of elective intervention is constant from conception until some months after delivery, and compression is offered throughout. Figure 3 shows both, plotting the gestational trajectory of venous load and thrombotic risk against the indicative timing of each management option.

Three caveats are load-bearing. The pregnancy-specific evidence beneath the framework is thin, the interventional evidence for any treatment amounting to seven trials in the Cochrane synthesis [8] plus three later small studies [45,71,95] with serial imaging studies enrolling between 8 and 66 women each [43,53]. No guideline identified here distinguishes first-, second- and third-trimester risk for any venous intervention, so the trimester structure is imposed on evidence not generated with it in mind. And no study reports paired CEAP classes in the same women in the third trimester and again postpartum, so the framework’s central assumption—that a substantial proportion of third-trimester findings will regress—rests on anatomical and physiological endpoints rather than a validated clinical trajectory.

### 8.2. Preconception to Delivery

Before conception, three conversations are worth having. On timing: since pregnancy is associated with recurrence after saphenous surgery [114] and with recurrent pelvic reflux after embolisation [115], and recurrence is common in any case [116], a woman intending further pregnancies should understand that treating now may need repeating. Treatment before conception carries no pregnancy-specific restriction and follows standard indications, individualised to the woman’s reproductive plans. On expectations: roughly one in four previously unaffected women reports new varicose disease during a pregnancy (Section 4.1) [57], most gestational change regresses [46,53], and family history is a stronger determinant than parity [57]. On documentation: a pre-pregnancy clinical and duplex record allows any later assessment to be made by direct comparison rather than by recall.

The first trimester is dominated by early hormonal and volume-related dilation, measurable before any mechanical effect of the uterus [24,51], with symptoms usually mild and thrombotic risk at its lowest (Table 1). The tasks are baseline documentation—risk factors, varix distribution including vulvar and perineal sites, bilateral calf circumferences for later comparison—and information on the effect of pregnancy on varicose veins, as NICE recommends [9]. Duplex is reserved for red flags, significant pre-existing disease, prior intervention and symptoms disproportionate to signs; routine screening of asymptomatic women is not supported. Conservative measures are introduced, including low-pressure compression for symptom relief, individualised according to symptoms, tolerability and clinical context; no evidence shows that starting earlier prevents varicose veins [94], but it allows a habit to form before donning becomes harder. Elective intervention is deferred [9,17] and venoactive drugs avoided [19,20,21,22].

The second trimester is when plasma volume expands fastest (Section 3.2), when most women first notice symptoms, and when vulvar varices characteristically appear (Section 7). Assessment adds specific enquiry about vulvar and perineal discomfort, seldom volunteered, and review of compression adherence; pressure should be reduced rather than increased if tolerance is limiting [94,99]. Duplex is indicated for new or worsening unilateral symptoms, suspected superficial vein thrombosis and atypical varix distribution suggesting a pelvic origin, although pelvic vein imaging has no validated role in pregnancy [67]. NSAIDs are avoided from 20 weeks [23]. Where symptoms remain limiting despite optimised compression the options are few: routine initiation of a venoactive agent is not supported, and any individual licenced product considered is governed by the labelling and national licencing set out in Section 6.1 [19,20,21,22].

The third trimester is the period of maximal load: every change quantified in Section 3 and Table 1 is at or near its peak, and reflux, where it develops, appears predominantly now [46]. Superficial vein thrombosis incidence and symptom burden reach their antenatal peak, and diagnostic algorithms perform least efficiently [28,29]. We therefore suggest a low threshold for duplex when a red flag is present, since both venous disease and thrombosis are most likely now and clinical discrimination least reliable, but not for the aching, heaviness and dependent oedema that are near-universal at this gestation. Standing examination is often poorly tolerated at this stage. Prolonged supine positioning is inadvisable because of aortocaval compression [26,47]. The symptomatic repertoire reduces to compression, elevation, left lateral rest and paracetamol, and gross or symptomatic varicose veins with phlebitis, oedema or skin change should reach the obstetric team as a scoreable venous thromboembolism risk factor [93].

At delivery, vulvar varices are not in themselves a contraindication to vaginal birth. The published case material cannot support a comparative estimate of risk between modes of delivery, and no denominator exists for rupture risk (Section 7), the stated exception in the primary clinical series being markedly dilated vaginal veins of 1 cm or more [118]. We suggest that mode of delivery be decided on obstetric grounds with vascular input.

### 8.3. Early Postpartum and Definitive Reassessment

In the early puerperium, venous distension and oedema begin to resolve as uterine compression is removed and cardiac output returns towards baseline [25], while thrombotic risk peaks: superficial vein thrombosis incidence reaches its gestational maximum (Table 1) and postpartum venous thromboembolism incidence is several-fold higher than antepartum [28,123,124]. This period is therefore about vigilance rather than venous management. Persistent or new unilateral leg symptoms, a tender superficial cord, and postpartum fever with abdominal or flank pain require prompt evaluation. Compression may be continued for comfort and thromboprophylaxis follows obstetric risk assessment [93], but we suggest deferring definitive venous assessment, since regression is incomplete at this stage and a classification made now is likely to overstate persistent disease. Guidance on lactation after sclerotherapy is discrepant—polidocanol labelling advises considering interruption of breastfeeding for up to 8 h [110] while a European manufacturer’s product information, as reported in a German expert review, advises 2–3 days [31]—and the discrepancy should be disclosed rather than resolved silently.

Reassessment separates transient gestational change from established disease, but published thresholds for its timing differ by a factor of four. The NICE guideline development group recorded a general consensus, explicitly labelled as such, that intervention should follow at least 3–6 months after delivery [96]; a German review notes that varicosities and symptoms often regress within 3–6 months [31]; a review of vulvar varicosities suggests intervention if symptoms persist beyond 6–8 weeks postpartum [119]. The only observational time-course data concern vulvar varices (Section 7). Given this spread, we propose a pragmatic 3–6-month postpartum reassessment interval, or later if breastfeeding continues, with earlier review for persistent symptoms, complications or a clear treatment indication. This interval is author-derived and consensus-informed rather than biologically validated: no study has compared reassessment intervals against any clinical outcome. Reassessment comprises standing clinical examination, CEAP classification with the date recorded [66], and duplex mapping of the superficial and deep systems with attention to non-saphenous and pelvic-origin sources [73,76,122]. Once the constraint of pregnancy is removed, standard guidance applies [6,9,75,117]. Where further pregnancies are planned, recurrence is more likely [114,115,118], which is a reason to weigh timing rather than to withhold treatment from a symptomatic woman.

## 9. Limitations and Evidence Gaps

A narrative synthesis was preferred to a scoping or systematic review because the material to be integrated is heterogeneous in kind rather than merely large in volume. No single answerable question of the population–intervention–comparison–outcome (PICO) type governs that problem, and the outputs sought here rest on interpretive judgements that a screening-and-charting procedure does not make. The design carries its own limitations. The literature search was targeted rather than intended as an exhaustive retrieval, and the selection and weighting of sources rest on the authors’ judgement, so selection bias cannot be excluded. The safeguards described in Section 2.4, Section 2.5 and Section 2.6 make that judgement visible but do not remove the influence of selection. The underlying literature imposes further constraints. Pregnancy-specific studies are few, small and heterogeneous, several of the classic studies are three decades old, and the major contemporary interventional trials cited here formally excluded pregnant women [112,113], so extrapolation is unavoidable rather than optional in the therapeutic sections.

Each absence of evidence reported here is an observed absence in accessible literature rather than a demonstrated non-existence. Two further limitations deserve explicit statement. First, the trimester-stratified framework of Section 8 has not been prospectively validated, tested against clinical outcomes or put through a formal consensus process. Second, this is not the first review to address the field: Mitschang and Görge have set out how pregnancy alters phlebological treatment decisions [31], and the clinical substance overlaps substantially. What is added here is organisation by gestational stage rather than by intervention, an explicit and consistently applied separation of pregnancy-specific from extrapolated evidence, integration of the diagnostic pitfalls with the management sequence, and specification of the research gaps at the level of study design; no claim of conceptual novelty is made.

## 10. Future Work

The research needs set out here are ordered by the importance of the question rather than by the ease of answering it.

A prospective longitudinal cohort spanning preconception to at least one year postpartum is the single most important missing study: no study identified here has followed the same women from before conception through gestation into late postpartum with paired clinical classification and duplex. An adequately powered prospective longitudinal cohort, enrolling women with and without pre-existing venous disease and applying standardised duplex at fixed gestational windows and at defined postpartum intervals with CEAP classification dated at each visit, would address the incidence of genuinely new disease, the proportion of gestational change that reverses, the determinants of persistence and the cumulative effect of parity.

Pregnancy-specific duplex reference values are needed because current reflux thresholds [72] and diameter norms were derived in non-pregnant subjects; whether Valsalva and distal augmentation behave equivalently against a chronically raised intra-abdominal pressure is also unstudied. A normative study of gestational-age-specific reflux duration and vein diameter by elicitation manoeuvre would give third-trimester findings the right comparator.

A pregnancy compression trial with clinical endpoints is overdue, existing trials being small, incompletely blinded and limited to symptom or surrogate outcomes [45,71,94,95]. An adequately powered randomised trial with credible sham control, validated symptom and quality-of-life outcomes, prespecified postpartum assessment of persistent disease and objectively reported adherence is needed; a design comparing class I with class II garments would also address the dose question, which currently rests on a within-woman comparison in 27 women [94]. Validation of outcome measures in pregnancy is a prerequisite for any such trial, since no venous-specific patient-reported outcome measure has been validated in pregnant women [69,70].

Four further gaps are tractable. Predictors of persistence would allow reassessment to be targeted, affordable candidates including family history, parity, pre-pregnancy venous status, third-trimester saphenous diameter and calf pump function. Prospective studies comparing clinically relevant postpartum reassessment intervals are needed to determine when gestational venous change has regressed sufficiently for stable classification and treatment planning; the published thresholds are consensus-based and have never been compared against any clinical outcome [96,119]. Superficial vein thrombosis in pregnancy needs anatomical and treatment data, since the largest cohort establishes incidence and prognosis but records no anatomical detail [28] and treatment rests on a conditional, low-certainty recommendation [78]; a prospective registry of thrombus extent, junction distance, treatment and outcomes would define who requires anticoagulation, at what dose and for how long, questions a randomised trial here is unlikely ever to answer directly. Finally, vulvar and pelvic-origin disease rests on clinic series for prevalence, a single small cohort for the postpartum time course, and no denominator at all for the rupture risk driving mode-of-delivery decisions [118,119,120]. Interventional evidence for exceptional indications can come only from a registry.

Two further needs are of a different kind. Pregnancy-specific safety evidence for venoactive agents is unlikely to come from randomised trials conducted in pregnancy, which would be difficult to justify for a symptomatic indication; exposure registries and pharmacovigilance linkage could nonetheless establish whether the preparations already taken in early gestation, often before pregnancy is recognised, carry measurable risk [19,20,21,22]. And the framework proposed in Section 8 has not itself been evaluated: prospective application in a defined antenatal population, recording whether the epoch-specific actions are feasible, whether they alter imaging use or referral, and whether the proposed reassessment interval identifies persistent disease, would establish whether it is useful.

## 11. Conclusions

Pregnancy is a dynamic venous state rather than a static risk factor. The venous system dilates, slows and gains capacitance from early gestation and most of that change reverses after delivery, although an incompletely reversible superficial residue remains contested. New reflux develops in a minority of previously competent limbs and was undetectable postpartum in the one study reporting it, and ambulatory venous hypertension was not demonstrated in the pregnancy studies that assessed it.

Physiological gestational change, chronic venous disease and thrombotic pathology overlap clinically, and the principal hazard is failure to separate them. Lower-limb venous duplex resolves much of that uncertainty and involves no foetal insonation at any gestational age, but should answer a question that changes management. A venous abnormality first detected in pregnancy warrants postpartum reassessment before it is taken for persistent structural disease; this does not apply to women entering pregnancy with established chronic venous disease.

Pregnancy-specific evidence remains limited, and evidence extrapolated from non-pregnant populations should be identified as such rather than applied unexamined. Management is accordingly conservative: compression can relieve symptoms and limit oedema but has not been shown to prevent varicose veins, routine initiation of venoactive drugs is not supported, and elective intervention is deferred other than where clinically necessary, although specialist assessment should not be. For the clinician the practical consequences are to treat symptoms rather than duplex findings, to keep the threshold for objective imaging low when a red flag is present and high for the aching and dependent oedema that are near-universal in late gestation, and to record any antenatal classification as provisional and dated. The trimester-stratified framework proposed here arranges these decisions along the gestational axis; it is author-derived and awaits prospective validation.

## Figures and Tables

**Table 1:** Principal venous changes arising during pregnancy, their peak timing and their reversibility. The table describes changes developing in gestation; it does not address women entering pregnancy with established chronic venous disease. The Evidence column states the source population in which each observation was generated, using the convention applied throughout this review: PH, pregnant human; NPH, non-pregnant human; A, animal; IV, in vitro. Every entry in this table is PH. These source-population labels are distinct from the [PS]/[EX]/[CV] labels used in Supplementary Tables S3 and S4, which describe how a recommendation is supported rather than where an observation was made. Neither set is a certainty-of-evidence grade: both describe provenance only. The full mechanism-by-mechanism account, with quantitative detail and source population for every entry, is given in Supplementary Table S1.

Change	Peak Timing	Reverses After Delivery?	Evidence
Plasma volume +42–48% above baseline	Third trimester	Yes	PH; meta-analysis, 347 pregnancies [24]
Cardiac output +31%	Early third trimester	Yes	PH; pooled, 39 studies [25]
Venous distensibility increased	Throughout	Yes (converges postpartum)	PH; plethysmography [33,41]; one discordant study [42]
Common femoral +22%, saphenofemoral junction +48%	Late third trimester	Yes	PH; serial duplex, 8 women [43]
Great saphenous diameter peak	≈34 weeks	Largely; incomplete on the left in one study	PH; serial duplex [27,53]
Popliteal velocity and flow reduced ≈ one third	Late pregnancy	Yes	PH; controlled study [45]
Calf pump dysfunction in 17%	Third trimester	Yes, by 3 months	PH; two studies disagree [35,46]
New reflux in 0–15%	Third trimester	Usually; none postpartum in the one study reporting it	PH; three studies, genuinely divided [27,43,46]
Superficial venous residue	Postpartum	Contested—one study reports incomplete reversal	PH; separate nulliparous comparison group [51]
Ambulatory venous hypertension	Not demonstrated in the pregnancy studies that assessed it	Not applicable	PH; assessed in two studies [43,46]
Superficial vein thrombosis incidence 0.1 → 1.6 per 1000 person-years	Postpartum	Risk returns to baseline	PH; nationwide cohort [28]

**Table 2:** Trimester-stratified framework at a glance. The framework is author-derived, evidence-informed and hypothesis-generating: it is not a clinical guideline or formal consensus recommendation and should not be cited as one. It combines published evidence, guideline or regulatory positions and author-derived proposals, and has not been prospectively or externally validated or tested against clinical outcomes. Labels in the Priority action column state how each recommendation is supported: [PS] pregnancy-specific, resting on data generated in pregnant or postpartum women; [EX] extrapolated, resting only on data generated outside pregnancy; [CV] convention, where no directly applicable data were identified. An unlabelled priority action may reflect mixed support that no single label can represent. Entries in the Deferred column rest on guideline convention or regulatory restriction unless labelled otherwise. These labels describe provenance, not quality or certainty. CEAP, Clinical–Etiological–Anatomical–Pathophysiological classification; NSAID, non-steroidal anti-inflammatory drug. The full framework is given in Supplementary Table S4.

Epoch	Dominant Physiology	Priority Action	Deferred
Preconception	Substrate fixed; family history outweighs parity	Document clinical status and duplex [CV]; counsel on recurrence if further pregnancies are planned [PS]	No pregnancy-specific restriction [CV]; treatment may be considered according to standard indications and individual reproductive plans
First trimester	Early hormonal and volume-related dilation, before uterine compression	Baseline documentation and information-giving [CV]; compression at the lower pressure range may be offered for symptom relief [PS]	All elective intervention; routine initiation of venoactive drugs
Second trimester	Fastest plasma volume expansion; vulvar varices appear	Directed enquiry about vulvar symptoms [PS]; review compression adherence [EX]	Elective intervention; NSAIDs from 20 weeks; routine initiation of venoactive drugs; pelvic vein imaging, which has no validated role in pregnancy
Third trimester	Maximal load; reflux appears if it is going to; diagnostic algorithms least efficient	Prompt objective investigation of red flags; routine imaging of typical bilateral symptoms is generally unnecessary [CV]; modified examination posture [PS]	Elective intervention; NSAIDs; initiation of venoactive agents
Delivery	Vulvar varices in a minority; rupture risk has no denominator	Decide mode of delivery on obstetric grounds with vascular input [CV]	Prophylactic caesarean section on venous grounds alone
Early postpartum (to ≈6 weeks)	Distension resolving; thrombotic risk at its peak	Vigilance for thrombosis, not venous management [PS]	Definitive assessment and classification, which would overstate persistent disease at this stage [PS]; all elective intervention
Late postpartum (pragmatic ≈3–6-month interval)	Most gestational change has regressed; persistent disease becomes assessable	Standing examination, dated CEAP, duplex mapping [CV]	Management can generally transition toward standard non-pregnancy venous guidance once postpartum physiological regression has been reassessed

## Data Availability

No new data were created or analysed in this study. All data discussed are available in the cited publications.

## Supplementary Materials

The following supporting information can be downloaded at: https://www.mdpi.com/article/10.3390/jcm15208111/s1; Text S1: Literature Search: Sources, Search Period, and Search Terms; Table S1: Pregnancy-related venous changes, their gestational pattern and clinical implications; Table S2: Diagnostic challenges in chronic venous disease during pregnancy and the puerperium; Table S3: Management options for chronic venous disease during pregnancy and the postpartum period; Table S4: Proposed trimester-stratified framework for the assessment and management of chronic venous disease in pregnancy and the puerperium; Table S5: Mapping of the SANRA items to the sections of this review in which they are addressed.

## Abbreviations

A, animal (evidence source population); ACOG, American College of Obstetricians and Gynecologists; AIUM, American Institute of Ultrasound in Medicine; ASH, American Society of Hematology; AVF, American Venous Forum; AVLS, American Vein and Lymphatic Society; AWMF, Association of the Scientific Medical Societies in Germany; CEAP, Clinical–Etiological–Anatomical–Pathophysiological classification; CI, confidence interval; CT, computed tomography; CV, convention (evidence label); CVD, chronic venous disease; DVT, deep vein thrombosis; EMA, European Medicines Agency; ESC, European Society of Cardiology; ESVS, European Society for Vascular Surgery; EX, extrapolated (evidence label); FDA, United States Food and Drug Administration; GSV, great saphenous vein; GWAS, genome-wide association study; HR, hazard ratio; IV, in vitro; LEFt, Left leg–Edema–First trimester clinical decision rule; LMWH, low-molecular-weight heparin; MR, magnetic resonance; NICE, National Institute for Health and Care Excellence; NPH, non-pregnant human; NSAID, non-steroidal anti-inflammatory drug; PE, pulmonary embolism; PH, pregnant human; PICO, population, intervention, comparison, outcome; PP, postpartum; PS, pregnancy-specific (evidence label); RCOG, Royal College of Obstetricians and Gynaecologists; RR, relative risk or risk ratio; SANRA, Scale for the Assessment of Narrative Review Articles; SEM, standard error of the mean; SOMANZ, Society of Obstetric Medicine of Australia and New Zealand; SVP, Symptoms–Varices–Pathophysiology classification of pelvic venous disorders; SVS, Society for Vascular Surgery; SVT, superficial vein thrombosis; VTE, venous thromboembolism.
