# Supplementary material for: Chronic Venous Disease in Pregnancy: Pathophysiology, Diagnostic Pitfalls, and a Trimester-Stratified Management Framework—A Narrative Review
Source: J Clin Med. 2026 Oct 19;15(20):8111. doi: 10.3390/jcm15208111 (PMC13706563; doi:10.3390/jcm15208111)
Supplement: Supplementary file 1 [file jcm-15-08111-s001.zip › jcm-4550470-supplementary.pdf]

Supplementary Materials

Chronic Venous Disease in Pregnancy: Pathophysiology, Diagnostic Pitfalls, and a Trimester-Stratified Management Framework—A Narrative Review

Text S1 describes the literature search. Tables S1–S4 give the full evidence detail underlying the manuscript: Table S1 expands Table 1; Table S2 supports Section 4 and Figure 2; Table S3 supports Section 6; Table S4 expands Table 2 and Figure 3. Reference numbers correspond to the reference list of the main manuscript. Table S5 maps the SANRA items to the sections of the manuscript in which they are addressed.

Text S1. Literature Search: Sources, Search Period, and Search Terms

This narrative review was based on a targeted literature search directed at the clinical and mechanistic questions defined in Section 1 of the manuscript. This text describes the information sources, the search period, the principal search concepts and terms, the way in which the concepts were combined and the types of literature considered.

Information sources

PubMed/MEDLINE and Europe PMC were searched. The Cochrane Library, the NICE evidence repository and the websites of guidance-issuing bodies (ESVS; SVS, AVF and AVLS; RCOG; ACOG; ASH; ESC; SOMANZ; AWMF; EMA; FDA) were consulted directly, and Crossref and OpenAlex were used for record lookups. Reference lists of retrieved articles were checked for additional relevant sources.

Search period

Searching began in January 2026 and was last updated on 20 August 2026. No lower date limit and no language restriction were applied.

Search concepts and terms

Four concepts were combined. The principal terms for each concept are listed below; they are representative of the terms used rather than a verbatim record of individual queries.

Venous disease: varicose veins; chronic venous disease; chronic venous insufficiency; venous reflux; superficial vein thrombosis; deep vein thrombosis; vulvar varices; pelvic varices.

Pregnancy and the puerperium: pregnancy; pregnancy trimesters; postpartum period; puerperium.

Assessment: duplex ultrasonography; Doppler ultrasonography; CEAP; venous severity scoring; plethysmography; venous pressure.

Management: compression stockings; venoactive agents; phlebotonic agents; sclerotherapy; endovenous ablation; phlebectomy; anticoagulation.

Combination of concepts

Venous-disease terms were combined with pregnancy and puerperium terms and, according to the question being addressed, with assessment or management terms, as in the following illustrative example:

> (varicose veins OR venous reflux OR superficial vein thrombosis) AND (pregnancy OR puerperium OR postpartum period) AND (duplex ultrasonography OR compression stockings)

Types of literature considered

Guidelines, consensus statements and regulatory or product documents; systematic reviews and meta-analyses; randomised trials; observational, diagnostic-accuracy and physiological studies; seminal mechanistic work; and case series or case reports where no higher-level pregnancy evidence was identified. The scope of the review and the prioritisation of sources, including the precedence given to evidence generated in pregnant or postpartum women, are described in Sections 2.3 and 2.4 of the manuscript. The resulting narrative synthesis draws on the 124 sources cited in the manuscript.

Table S1. Pregnancy-related venous changes, their gestational pattern and clinical implications. Evidence population indicates where the data were generated: PH, pregnant human; NPH, non-pregnant human; A, animal; IV, in vitro. These labels describe the source population of a mechanistic observation and are distinct from the [PS]/[EX]/[CV] labels in Table S4, which describe how a recommendation is supported. Neither set is a certainty-of-evidence grade: both describe provenance only.

Physiological change	Gestational pattern	Clinical implication	Evidence population	Key references
Progesterone-mediated venous smooth muscle relaxation	Presumed from early gestation; not directly measured in pregnant women	Offered as the explanation for early dilation preceding any mechanical effect; unconfirmed	NPH/IV (receptors in 90% of saphenous specimens); PH (no correlation with calf pump function)	[34,35]
Oestrogen contribution to wall compliance	Rises across gestation	Frequently cited, weakly supported; internally inconsistent receptor data	NPH (receptor and menopausal association studies); A (ovariectomy)	[34,36–38]
Relaxin-mediated vasodilation	Corpus luteum secretion from early pregnancy	Often invoked for venous dilation without direct evidence	A and PH, but exclusively arterial and renal	[39]
Matrix metalloproteinase-mediated matrix degradation	Not measured in pregnancy	Plausible molecular route from hormonal milieu to wall remodelling	NPH/IV only; no gestational vein-wall data identified	[40]
Plasma volume expansion	≈6% (first), 18–29% (second), 42–48% (third); fastest in the first half of the second trimester	Explains why symptoms commonly begin mid-gestation	PH (meta-analysis, 10 longitudinal studies, 347 pregnancies)	[24]
Cardiac output	Non-linear rise peaking in the early third trimester, returning to baseline postpartum	Peak circulatory load coincides with peak symptom burden	PH (pooled across 39 studies)	[25]
Venous distensibility and tone	Greater in pregnancy than postpartum ($P < 0.01$); reduced in pre-eclampsia in proportion to severity	Supports an actively regulated rather than passively overloaded venous compartment	PH (plethysmography); complicated by a 9-woman study showing increased tone	[33,41,42]

Physiological change	Gestational pattern	Clinical implication	Evidence population	Key references
Venous filling index and junctional diameter	Rise to the late third trimester, return postpartum; all values remain within the normal range	Antenatal duplex indices are not interpretable against non-pregnant norms	PH (serial study, 8 women)	[43]
Flow velocity and volume	Popliteal velocity 6.32 ± 0.28 vs 9.14 ± 0.37 cm/s and flow 118.48 ± 8.83 vs 177.73 ± 11.74 mL/min vs non-pregnant ($P < 0.0001$); lower still in oedematous legs	Quantifies the stasis limb of Virchow's triad; links benign venous change to thrombotic risk	PH (non-randomised controlled study, late pregnancy)	[44,45]
Calf muscle pump function	Pathological in 17% of 24 normal pregnancies in one study (reported range 4.7–37%, interval type not stated in the source); unchanged throughout in another	Symptoms track pump abnormality and resolve postpartum; studies disagree	PH (both)	[35,46]
Iliocaval compression by the gravid uterus	Progressive from mid-gestation; maximal at term; position-dependent	Basis for left lateral rest and for modified examination posture in late pregnancy	PH (venography, serial duplex); NPH (anatomical substrate)	[26,44,47,48,50]
Superficial vein dilation	Detectable in early pregnancy, before uterine compression is possible; great saphenous diameter peaks ≈34 weeks	Mechanical obstruction cannot be the initiating mechanism	PH (serial duplex)	[27,51]
New venous reflux	Absent in two serial studies; 5% (second trimester) rising to 15% (third), none postpartum, in a third	Third-trimester reflux should not be assumed permanent; evidence is genuinely divided	PH (all three)	[27,43,46]
Reversibility of dilation	Deep and junctional dilation reverses completely; superficial diameters return to first-trimester values; one study reports an incompletely reversible superficial residue	Persistence should be reassessed postpartum before a newly detected abnormality is interpreted as permanent structural disease	PH (serial); the dissenting study used a separate nulliparous comparison group	[43,46,51,53]
Ambulatory venous hypertension	Not demonstrated in the pregnancy studies that assessed it	The venous hypertension model of conventional CVD should not be transferred wholesale to normal pregnancy	PH; NPH for the model itself	[43,46,52]
Gestational oedema	Multifactorial; fall in colloid osmotic pressure insufficient to explain it	Oedema carries little diagnostic information about the venous system	PH	[54]
Pre-existing genetic substrate	Fixed before conception	Determines who dilates, refluxes and progresses; family history outperforms parity as a clinical predictor	NPH (monogenic and genome-wide association evidence); PH (family-history cohorts)	[5,55–58]
Parity	Cumulative exposure across pregnancies	The most frequently repeated and least consistently demonstrated risk factor	Mixed; supporting and opposing datasets are both large	[7,59–63]
Pelvic and ovarian venous reflux	Dilated incompetent ovarian veins common in parous asymptomatic women (63% vs 10% nulliparous, $P < 0.05$)	May explain a parity effect without change in saphenous anatomy	NPH	[63,64]

Table S2. Diagnostic challenges in chronic venous disease during pregnancy and the puerperium. Quantitative values are given with their sources; the assessment column states what should be done, not what may be inferred.

Clinical finding	Physiological explanation	Pathological differential	Red flags	Appropriate assessment
Bilateral dependent leg oedema	Plasma volume expansion, reduced colloid osmotic pressure, iliocaval compression [12,24,26,54]	Venous oedema; pre-eclampsia; lymphoedema; cardiac, renal or hepatic causes	New, rapid or facial oedema; hypertension, headache or visual symptoms	Clinical assessment; blood pressure and proteinuria if pre-eclampsia is considered [87,88]; venous imaging is usually not required for a typical bilateral dependent pattern in the absence of red flags or atypical features
Unilateral leg swelling	Not adequately explained by pregnancy physiology	Deep vein thrombosis, left-sided or bilateral in 82.2% (95% CI 75.1–87.5) of pregnancy cases [14]; most are proximal and spare the calf [13]	Any new unilateral swelling, particularly left-sided; calf circumference difference ≥2 cm	Whole-leg compression duplex including iliac veins; serial imaging or MR venography after a negative initial study [78]; single complete study leaves a 3-month VTE rate of 1.1% (95% CI 0.3–4.0%) [79]
Leg aching, heaviness, nocturnal cramps, restlessness	Near-universal in late gestation; restless legs syndrome affects 22.9% (95% CI 14.7–33.8%) in the third trimester [11]	Chronic venous disease; restless legs syndrome; musculoskeletal causes	Symptoms disproportionate to visible signs	History and examination; duplex only if disproportionate, unilateral or atypical
Visible varicosities	Superficial dilation, maximal in the third trimester and largely reversible [43,53]	Established varicose disease; pelvic-origin varices	Tender, indurated, erythematous cord	Duplex is not required to confirm that visible varicose veins are varicose; it is required for suspected superficial vein thrombosis [28,89]
Tender indurated superficial vein	Not physiological	Superficial vein thrombosis: incidence rises 0.1 → 1.6 per 1000 person-years from first trimester to postpartum; 10.4% subsequently develop VTE (HR 83.3, 95% CI 46.3–149.7) [28]	Extension towards a junction; concurrent deep symptoms	Duplex documenting extent, junction distance and deep system; concurrent DVT/PE in ≈25% of general-population cases [89]
Reflux duration marginally beyond threshold	Thresholds (>0.5 s superficial/calf, >1.0 s femoral/popliteal) derived in non-pregnant subjects [72–74]; diameters rise ≈22% (common femoral) and ≈48% (saphenofemoral junction) [43]	Genuine reflux; physiological gestational dilation	None in isolation	Report with gestational age; describe rather than classify; defer definitive CEAP class and treatment planning to postpartum reassessment
Radiological left common iliac vein compression	Present in many asymptomatic non-pregnant adults [48]	May-Thurner physiology; iliofemoral thrombosis	Acute severe leg or buttock pain with swelling	Demonstration alone establishes neither causation nor an indication for intervention; exclude thrombosis [13]
Raised D-dimer	Physiological; essentially no healthy woman remains below 0.50 mg/L by the third trimester [80]; the 95th centile exceeds the non-pregnant cut-off throughout gestation [81]	Venous thromboembolism	Symptoms of PE: dyspnoea, pleuritic pain, haemoptysis, syncope, unexplained tachycardia	Use only within a validated pathway at algorithm-specific thresholds [29,85]; guidance is divided on any use in pregnancy [82–84]
Absence of clinical decision-rule criteria	LEFt rule specificity only 32% in external validation	Thrombosis not excluded: upper confidence bound 7.9% among women with no criteria [16]; two of three criteria are produced by normal gestational change [15]	—	Do not use as a standalone rule-out; imaging where clinical suspicion persists
Vulvar, perineal or gluteal varices	Pelvic venous congestion; appear at 18–24 weeks in a first pregnancy [118]	Pelvic venous disorder; pelvic-origin leg varices	Thrombophlebitis; bleeding	Directed inspection, which is required since these are rarely volunteered; CEAP does not classify them, so use the SVP instrument alongside it [67]
Postpartum fever with abdominal or flank pain	Not physiological	Ovarian vein thrombosis; septic pelvic thrombophlebitis	Any occurrence	Cross-sectional imaging, not leg duplex [91,92]

Clinical finding	Physiological explanation	Pathological differential	Red flags	Appropriate assessment
Concern about fetal safety of imaging	Restrictive Doppler statements are scoped to embryonic or fetal insonation [77]	—	—	Lower-limb venous duplex involves no fetal insonation and may be performed at any gestational age; where thoracic imaging is indicated, fetal doses from scintigraphy and CT pulmonary angiography are low [86]

Table S3. Management options for chronic venous disease during pregnancy and the postpartum period. Evidence basis: PS, supported by data generated in pregnant or postpartum women; EX, supported only by data generated outside pregnancy; CV, no directly applicable data identified, resting on guideline convention or expert opinion. These labels describe the provenance of the supporting evidence, not its quality or certainty, and are distinct from the PH/NPH/A/IV source-population labels of Table S1.

Intervention	Pregnancy-specific evidence	Expected benefit	Safety and limitations	Appropriate timing	Evidence basis
Low-pressure graduated compression (18–21 mmHg/class I in the cited pregnancy trial)	Four small randomised or quasi-randomised trials [8,71,94,95]; no significant difference in outcome between class I and class II in an intra-individual comparison [94]. No pregnancy-specific dose-finding study has established an optimal compression pressure	Symptom relief and limitation of oedema	Does not prevent varicose veins [94,96]; adherence limited by discomfort and donning difficulty [95,99,100]; the magnitude of the reported effects warrants cautious interpretation given the small sample sizes, incomplete blinding and reliance on subjective or surrogate outcomes	Throughout pregnancy and postpartum for comfort	PS
Higher-pressure compression (>21 mmHg)	No pregnancy dose-finding study; non-pregnant dose–response data only [98]	No demonstrated additional benefit in pregnancy	Poorer tolerance and greater application difficulty [99]	Not routinely; reduce rather than increase pressure if tolerance is limiting	EX
Anti-embolism stockings, 14–15 mmHg calf	Recommended for hospitalised pregnant or postpartum women with a contraindication to LMWH [93]	Thromboprophylaxis, not venous symptom control	A different device, pressure and indication from therapeutic hosiery; the two literatures should not be merged	Hospitalisation with a contraindication to LMWH	PS (thromboprophylaxis indication)
Leg elevation, avoidance of prolonged standing, calf activation	Indirect; calf pump physiology and general-population risk-factor data [3,59,103]	Symptom relief; plausible reduction in venous stasis	Pregnancy-specific venous outcome data are limited; these measures are generally regarded as low-risk supportive strategies	Throughout	EX
Physical activity (≥150 min/week moderate aerobic)	General antenatal recommendation [102]; venous benefit inferred	General health; plausible venous benefit	Venous endpoint not demonstrated	Throughout	EX
Water immersion; Buerger–Allen exercises	Small pregnancy studies [8,104,105]	Short-term reduction in leg volume and oedema-related pain	Small, mostly unblinded; benefit not durable or disease-modifying	Second and third trimesters for symptomatic oedema	PS (small, mostly unblinded studies)
Rutosides	One trial of 69 women from 28 weeks: symptom improvement RR 1.89 (95% CI 1.11–3.22), moderate quality [8,31]	Historical symptom-relief signal; not established for routine use	Pregnancy-specific safety data insufficient; first-trimester use not recommended by product labelling [22]; single small trial reported decades ago; national licensing differs materially	Routine initiation not supported at any gestation; any use is a product-specific, individualised decision	PS (efficacy signal only); safety evidence absent

Intervention	Pregnancy-specific evidence	Expected benefit	Safety and limitations	Appropriate timing	Evidence basis
Other venoactive agents (horse chestnut, *Ruscus*, red vine leaf, calcium dobesilate)	None	Extrapolated symptom and oedema reduction [106,107]	EU herbal monographs state that safety in pregnancy and lactation has not been established and that use is not recommended in the absence of sufficient data [19–21]; this records absence of study rather than observed harm, and is neither a contraindication nor reassurance. Calcium dobesilate carries a rare agranulocytosis association	Routine initiation not supported at any gestation	EX (efficacy); safety evidence absent
Paracetamol	National position statement supports use when medically indicated [108]	Analgesia	Lowest effective dose, shortest duration	Throughout	PS
NSAIDs	Regulatory advice against use from 20 weeks [23]	Analgesia and anti-inflammatory effect	Oligohydramnios from fetal renal dysfunction; ductal closure from ≈30 weeks; the advice also covers topical NSAIDs other than those applied to the eye	Avoid from 20 weeks	PS (safety)
LMWH for proven superficial vein thrombosis	ASH conditional recommendation on low-certainty evidence; no agreement on dosing, general agreement among panel members on treatment for the remainder of pregnancy and 6 weeks postpartum [78]; a regional position paper proposes consensus-based regimens [30]	Reduction in thrombotic extension and VTE	Pivotal non-pregnant trial excluded junction-proximal thrombosis [109]; no validated pregnancy-specific junction-distance threshold	On diagnosis, after duplex	EX
Sclerotherapy	None	Not applicable during pregnancy	Relative contraindication in international consensus [18]; contraindicated in a European manufacturer's product information and the German sclerotherapy guideline, as reported in a German expert review [31]; US labelling cites limited benefit rather than demonstrated harm [110]; lactation guidance discrepant (8 h vs 2–3 days) [31,110]	Defer to a pragmatic ≈3–6-month postpartum interval, or after breastfeeding	CV
Endovenous thermal, mechanochemical or cyanoacrylate ablation	None; pregnancy was an exclusion criterion in the pivotal trials, and breastfeeding in one of them [112,113]	Not applicable during pregnancy	No guideline identified addresses these modalities in pregnancy specifically; tumescent anaesthesia never studied in pregnancy	Defer to a pragmatic ≈3–6-month postpartum interval	CV
Open surgery for varicose veins	None	Not applicable during pregnancy	Known pregnancy listed among absolute contraindications to elective operation [17]	Defer	CV
Intervention for bleeding varicosity	None specific to pregnancy	Haemostasis; prevention of re-bleeding	The recognised exception to deferral; elevation, direct	Immediate, at any gestation	CV

Intervention	Pregnancy-specific evidence	Expected benefit	Safety and limitations	Appropriate timing	Evidence basis
			pressure and compressive bandage, then urgent vascular referral [9,90]; consensus favours compression and sclerotherapy before suture ligation [117]		
Specialist vascular assessment	Required by German guidance for varices in pregnancy, variceal bleeding, superficial vein thrombosis or ulceration [17]; immediate referral of bleeding varicose veins under NICE guidance [9]	Correct characterisation; planning of postpartum treatment	Assessment should not be deferred merely because treatment will be	At presentation	CV

Table S4. Proposed trimester-stratified framework for the assessment and management of chronic venous disease in pregnancy and the puerperium. This table summarises a framework proposed by the authors; it was not derived from a formal consensus process, has not been prospectively or externally validated, and is not a guideline. It is author-derived, evidence-informed and hypothesis-generating, has not been tested against clinical outcomes, and should not be cited as a guideline or consensus recommendation. It combines published evidence, guideline or regulatory positions and explicitly identified author-derived proposals. Evidence labels as in Table S3: [PS] pregnancy-specific, [EX] extrapolated, [CV] convention. An unlabelled entry may reflect mixed support that no single label can represent. These labels describe the provenance of the supporting evidence, not its quality or certainty.

Gestational stage	Expected changes	Assessment priorities	Conservative management	Red flags and escalation	Interventions generally deferred
Preconception	Baseline substrate fixed; family history outweighs parity as a predictor [57]	Clinical and duplex documentation converting later assessment into comparison [CV]	Counselling on recurrence risk if further pregnancies are planned [PS] [114–116]	Existing advanced or post-thrombotic disease warrants vascular review before conception [CV]	No pregnancy-specific restriction; treatment may be considered according to standard indications and individual reproductive plans [CV]
First trimester	Early hormonal and volume-related dilation, measurable before uterine compression [24,51]; SVT incidence lowest, 0.1 per 1000 person-years [28]	Baseline record of risk factors, varix distribution including vulvar and perineal sites, bilateral calf circumferences [CV]; duplex only for red flags, pre-existing disease, prior intervention or symptoms disproportionate to signs [CV]	Information on the effect of pregnancy on varicose veins, as recommended by NICE [9]; activity [102]; avoidance of prolonged standing; low-pressure compression for symptom relief, individualised according to symptoms, tolerability and clinical context [PS] [94]	Unilateral leg swelling or pain, particularly left-sided, or calf circumference difference ≥2 cm; first-trimester presentation is itself a LEFt criterion [15,16]	All elective intervention [CV] [9,17]; routine initiation of venoactive drugs, which is not supported at any stage, with an explicit first-trimester labelling restriction for the one agent with pregnancy efficacy data [19–22]
Second trimester	Plasma volume expands fastest [24]; symptoms typically begin; vulvar varices appear at 18–24 weeks in a first pregnancy, 12–18 weeks subsequently [118]; SVT incidence 0.2 per 1000 person-years [28]	Directed enquiry about vulvar and perineal discomfort, seldom volunteered [PS] [118]; review of compression adherence [EX] [99]	Continue and re-measure compression; reduce rather than increase pressure if tolerance is limiting [PS] [94,99]; supportive garments for vulvar varices [118,119]; water immersion and calf exercise as adjuncts [8,104,105]	As first trimester, plus tender indurated superficial cord, and new or worsening oedema with hypertension, headache or visual symptoms [87,88]	Elective intervention [CV] [9]; NSAIDs from 20 weeks [23]; routine initiation of venoactive drugs [19–22]; pelvic vein imaging, which has no validated role in pregnancy [67]

Gestational stage	Expected changes	Assessment priorities	Conservative management	Red flags and escalation	Interventions generally deferred
Third trimester	Maximal load: cardiac output peaks [25]; plasma volume 42–48% above baseline [24]; iliocaval compression greatest [26]; saphenous diameter peaks ≈34 weeks [27]; popliteal velocity and flow reduced ≈one third [45]; reflux, where it develops, appears now [46]; SVT incidence 0.5 per 1000 person-years [28]; diagnostic algorithms least efficient [29]	Prompt objective investigation of red flags; routine imaging of typical bilateral symptoms is generally unnecessary in the absence of atypical features [CV]; modified examination posture, since prolonged standing and supine positioning are both problematic [26,47]	Compression, left lateral rest and paracetamol [PS]; leg elevation [EX]; referral of gross or symptomatic varicose veins to the obstetric team as a scoreable VTE risk factor [93]	As above, plus symptoms suggesting pulmonary embolism, requiring a validated pathway [29,83,85]	Elective intervention [9]; NSAIDs [23]; initiation of new venoactive agents [CV]
Delivery	Vulvar varices present in a minority; rupture risk has no published denominator [118,120]	Mode of delivery decided on obstetric grounds with vascular input, not on the appearance of varices [CV] [118,120]	Perineal support; documentation of varix distribution	Bleeding from a varicosity [9,90]	Prophylactic caesarean section on venous grounds alone, which the available case material cannot support [120]
Early postpartum (to ≈6 weeks)	Distension and oedema begin to resolve as cardiac output returns towards baseline [25]; thrombotic risk peaks [123,124]; SVT incidence 1.6 per 1000 person-years in the postpartum period (up to 12 weeks in the source study), ≈16 times the first-trimester rate [28]	Vigilance rather than venous management: persistent or new unilateral symptoms, tender superficial cord, postpartum fever with abdominal or flank pain requiring cross-sectional imaging [91,92]	Compression may continue for comfort; thromboprophylaxis follows obstetric risk assessment [93]	Any of the above; bleeding varicosity remains an indication for urgent referral [9,90]	Definitive venous assessment and classification, which would overstate persistent disease at this stage [PS]; routine elective definitive venous intervention is generally deferred while pregnancy-associated venous changes regress [CV], although bleeding varicosities, thrombotic complications and other clinically urgent indications are treated on their merits [9,90]; sclerotherapy, a relative contraindication in the postpartum and breastfeeding periods [18], with discrepant lactation-interruption advice [31,110]
Late postpartum (pragmatic ≈3–6-month interval, or after cessation of breastfeeding)	Most anatomical and haemodynamic change has reversed [43,46,53]; an incompletely reversible superficial residue is described [27,51]; vulvar varices resolve over a mean of 5.8 months with 20% persistence [118]	Definitive standing examination; CEAP classification with the date recorded [66]; duplex mapping with attention to non-saphenous and pelvic-origin sources [73,76,122]	Continue compression while awaiting treatment; counsel on recurrence before a further pregnancy [114,115,118]	Persistent symptoms, complications or a clear treatment indication warrant earlier review than the pragmatic 3–6-month interval [CV] [31,96,119]	Management can generally transition toward standard non-pregnancy venous guidance once postpartum regression has been reassessed [6,9,75,117]

Table S5. Mapping of the SANRA items to the sections of this review in which they are addressed. SANRA (Scale for the Assessment of Narrative Review Articles) comprises six items, each scored from 0 to 2 [32]; here, the items were used as a checklist during drafting and methodological review.

SANRA item	Where addressed	How it is addressed in this review
1. Justification of the article's importance for the readership	Section 1, paragraphs 1–4	Burden and definitional heterogeneity; pregnancy as a clinically distinct setting; the three features that make the problem clinically demanding; the variation of constraints across gestation.

SANRA item	Where addressed	How it is addressed in this review
2. Statement of concrete aims or formulation of questions	Section 1, final paragraph	Three stated aims, the gap identified, and the characterisation of the contribution as an evidence-organising synthesis of published evidence and guideline or regulatory positions, with a small number of explicitly identified author-derived proposals.
3. Description of the literature search	Section 2.2; Supplementary Text S1	Information sources, search period, principal search concepts and terms, an illustrative example of how the concepts were combined, and checking of reference lists; the scope of the review and the types of literature considered are described in Section 2.3.
4. Referencing	Throughout; reference list of 124 items	Quantitative statements are referenced to their primary sources, with the source population given in Table 1 and Table S1. Guideline, regulatory and product documents are named at the point of use.
5. Scientific reasoning	Sections 2.4–2.6; Sections 3–8	Source prioritisation as a preference rule rather than a certainty rating; qualitative judgement of transferability from non-pregnant populations; discordant findings retained and contextualised rather than reconciled; author-derived positions introduced as proposals and labelled [CV] where no directly applicable data were identified.
6. Appropriate presentation of data	Tables 1 and 2; Tables S1–S4; Figures 1–3	Quantitative values are given with confidence intervals or dispersion measures where the source reports them, and with the population in which they were generated.
